# Proceedings of the 92nd Annual Business Meeting of the American Association of Biological Anthropologists

**DOI:** 10.1002/ajpa.70147

**Published:** 2025-11-10

**Authors:** Amy L. Rector

**Affiliations:** ^1^ Anthropology, School of World Studies Virginia Commonwealth University Richmond Virginia USA

President Steve Leigh called the meeting to order at 6:30 p.m. Pacific Time on April 21, 2023. A quorum of over 40 Regular (voting) members present was established by the Secretary. The President welcomed attendees. All Annual Reports were uploaded on the AABA website prior to the Business Meeting and members were encouraged to review them online. The President, Vice President, Treasurer, *American Journal of Biological Anthropology* and *Yearbook of Biological Anthropology* reports were also presented orally at the meeting.

## 1 Report of the President

Steve Leigh presented comments and provided the following written report:

Please accept my appreciation and thanks to you, our members, for your involvement with and dedication to the Association. As usual, during our annual business meeting, we'll engage in the routine business of our association and celebrate the accomplishments of our outstanding colleagues with awards. Like last year, the business meeting will be broadcast live and recorded for those who are participating virtually this year.

The AABA has pursued numerous initiatives this year to address issues specific to the association as well as more general issues. Several initiatives have been substantial in their scope, involving tremendous commitment and hard work from many members. I thank all for their efforts and dedication to the discipline and to the AABA. We are a strong and innovative group, with a bright future.

### 1.1 Task Force on the Ethical Study of Human Materials

The Task Force on the Ethical Study of Human Materials has continued its extremely important work in the last year, and we thank co‐chairs Drs. Ben Auerbach and Fatimah Jackson for their sustained leadership. Details of the rationale and formation of the committee were discussed in last year's President's Report and during the Presidential Panel. The importance of the Task Force has only increased in the last year, with a survey completed in mid‐2022, as well as a preliminary survey report and a webinar with members of the Presidential Panel in late 2022. The report is available on the AABA website along with a recording of the webinar. The webinar included panelists from the conference event so that they could expand on the survey and address questions that emerged from the survey. The Task Force is continuing to move ahead on important issues, including coordination with granting agencies and cognate associations to increase our capabilities to address issues involved with our work and scholarship with human materials.

As noted last year, the Task Force decided to focus on the treatment of African American human materials across many domains. An objective of this work, in the long run, will be to help develop policies, guidelines, and best practices that apply broadly to matters involving human materials in research and teaching, as well as in other professional activities. Survey results indicate that a focus on African American issues aligns with member professional activities and interests, with nearly 45% of survey respondents reporting research or other scholarly activities involving African American remains. The initial survey results provide a strong baseline for the Task Force's efforts. In particular, the group anticipates greater engagement with leaders in the African American community, community‐based surveys, focus‐group‐based studies, and expanded community partnerships.

We envision the work of the Task Force as helping to set foundations for sustained advances on the part of the Association to engage positively with communities and to attend to ethical issues in all aspects of our work. This will involve continued work by the Committee on Community Partnership with the many communities with whom we work across our many subfields. In addition, we should expect the Association to develop carefully crafted policies, guidelines, and best practices that are sensitive to the needs and desires of communities in balance with scholarly practices, objectives, and prospects. An important element of this process will be a Presidential Panel at the Reno meetings. The Panel, co‐sponsored by the Committee on Diversity, Committee on Community Partnership, and the President, will explore community engagement in a broad sense. The panel brings together biological anthropologists from across our field to share their experiences, knowledge, and insights on this issue, with important implications for the Task Force's next steps and for our discipline more widely. The panel will help provide insights and resources for our members, and set foundations for future endeavors.

The Association has benefited tremendously from the sustained efforts and dedication of our Task Force co‐chairs, Drs. Jackson and Auerbach. In addition, we sincerely thank Task Force members who are also members of our Committee on Community Partnership (Drs. Shamsi Berry, Ellen Lofaro, Ripan Malhi, and Charlotte Roberts, chair Ben Auerbach). Additional members include Drs. Michael Blakey, Alan Goodman, Joseph Graves, Clarence Gravlee, and Sheela Athreya. Panelists from the 2022 meeting and webinar (Drs. Jennifer Caldwell, Carter Clinton, Joseph Jones, and Carmen Mosley) have graciously agreed to continue to engage with the Task Force. We express our deep appreciation and gratitude for their efforts and insights.

### 1.2 Annual Conferences

The 2022 conference in Denver was well‐received by attendees, and while we had fewer presentations and registrations than recent pre‐pandemic conferences, our time in Denver was productive and undertaken without major problems. Fortunately, we apparently did not see widespread problems with COVID‐19 (our reporting system received fewer than 10 positive reports following the conference). Despite this, we know that some members had considerable difficulty recovering from infections that were likely contracted in association with the conference. For 2023, we have continued COVID‐19 precautions, although with some easing from 2022.

This year, registration fees were increased over 2022 levels, in part to offset a modest deficit from the 2022 meetings of approximately $15,000. The deficit can be attributed to fewer registrations in 2022 than in previous years: a year with a typical number of registrations likely would not have resulted in a deficit. This indicates scaling effects of conference attendance, such that higher attendance results in lower marginal costs, greater revenue and thus both less risk and cost pressure for the Association. We are hoping that attendance rebounds to pre‐ pandemic levels with our 2024 conference in Los Angeles.

We have worked diligently to contain conference costs. For example, registration fees were held at 2019 levels through 2022, despite some inflationary cost pressures. However, given the modest deficit from 2022, I asked the Executive Committee to approve a small increase in registration fees on the assumption that 2023 abstracts and registrations would be comparable to 2022 levels. This measure was approved, and we implemented a fee structure for 2023 based on the fee structure in place for the 2020 Los Angeles meetings (which were not held in person). The increase—about $25 for regular members—was intended to cover a deficit on the order of the 2022 conference. Because abstract submissions in 2023 nearly matched 2022 levels and because fees increased slightly, we expect a “break even” year for our 2023 conference. It should be noted that the Association subsidizes conference costs through endowment income with the goal of keeping our member costs as low as possible. In fact, our registration fees remain remarkably low relative to peer and cognate organizations.

Our Vice President and Program Chair, Dr. Kristi Lewton, led extraordinary efforts throughout the year to conduct our 2023 virtual and in‐person conferences. I express my sincere gratitude to her. She has innovated throughout her first year in this position and carried out her responsibilities with noteworthy skill and goodwill, while helping to maintain the rigorous standards of our conference. Like 2022, this involved organizing both in‐person and virtual conferences. However, this year, Dr. Lewton was required to add the formidable task of selecting and contracting with a new virtual platform, following the demise of the company with which we worked in 2021 and 2022. Along with Dr. Lewton, we owe considerable thanks to colleagues on the Program Committee for their work in several areas, including abstract review and composing scientific sessions. Our Local Arrangements Committee, Drs. Marin Pilloud and Richard Scott, worked diligently with our Advance Team to help members plan all aspects of our meeting. Additional Advance Team members included Drs. Jonathan Bethard, Kristi Lewton, Leslea Hlusko, and Stephanie Meredith, and, from Burke and Associates, Lori Strong. The team was assisted by talented University of Nevada Reno graduate students, Ashley Baeza, Reecie Dern, Dori Kenessey, and Emily Smith.

### 1.3 Proposed Amendment to the AABA Constitution and By‐Laws

We have initiated a change to the AABA *Constitution and By‐Laws* regarding a section on the responsibilities of Local Arrangements Committees. Specifically, the proposed amendment concerns text stipulating responsibilities for Local Arrangements Committees (LAC). Historically, LACs had fiduciary and long‐term planning responsibilities for our annual conferences, including arranging hotel contracts, conference logistics, accounting, and other critical tasks. Those responsibilities are now handled by AABA officers along with our business partners at Burk and Associates Inc. Current LAC members continue to play essential scientific roles, assisting the Program Chair in organizing conferences while helping with general local matters. However, LACs no longer have financial, contractual, and logistical responsibilities.

The proposal to amend the document follows procedures specified by the AABA *Constitution and By‐Laws* (Article VII, Amendment). Specifically, five regular AABA members must initiate a change by submitting a request for amendment. Drs. Jamie Hodgkins, Caley Orr, Matthew Sponheimer, Michala Stock, and Anna Warrener, proposed the change, and we thank them for initiating the process. In line with our procedures, their proposal was reviewed and considered by the AABA Executive Committee, receiving unanimous approval. The next step is to discuss and consider this matter at our Annual Business Meeting and Awards Presentation. If the proposal is approved, a final vote will be conducted during our 2024 annual conference business meeting. If not, the *Constitution and By‐Laws* will remain unchanged, with no further consideration in 2024.

The current text of our *Constitution and By‐Laws* regarding the Local Arrangements Committee states:

Section 2b: Ad Hoc Committees. Each annual meeting of the Association shall be managed by a Local Arrangements Chair appointed by the President, with the approval of the Executive Committee, to serve for a term determined by the President. The Local Arrangements Chair is responsible for planning the meeting, overseeing and managing its operations and finances, bringing them to a conclusion, giving a final accounting, and reporting on these activities to the President, the Executive Committee, and the business meeting of the Association. Each Local Arrangements Chair is empowered to appoint an ad hoc Local Arrangements Committee to assist in carrying out these duties. Since each meeting will ordinarily have its own Local Arrangements Chair, there may be several such Chairs serving concurrently. The President may, when necessary and with the consent of a majority of the members of the Executive Committee, appoint other ad hoc committees to deal with specific issues. Ad hoc committees exist for a period of up to 3 years. Additional years may be added by the President and Executive Committee.

The proposed text states:

Section 2b: Ad Hoc Committees. Each annual meeting of the Association shall be supported by a Local Arrangements Committee appointed by the President to serve until the conclusion of the annual meeting. The Local Arrangements Committee is responsible for supporting the Vice President and Program Chair with local matters related to the annual meetings. The President may, when necessary and with the consent of a majority of the members of the Executive Committee, appoint other ad hoc committees to deal with specific issues. Ad hoc committees exist for a period of up to 3 years. Additional years may be added by the President and Executive Committee.

According to our *Constitution and By‐Laws*, an affirmative vote will mean that the proposal will be presented at our 2024 meetings in Los Angeles for final deliberations. As noted, a negative vote terminates the process.

#### 1.3.1 Yearbook of Biological Anthropology

Drs. Sheela Athreya and Graciela Cabana will begin their terms as co‐editors of the *Yearbook of Biological Anthropology* following this year's conference in Reno. We express our deepest thanks to Dr. Lyle Konigsberg upon completion of his term for guidance of the journal.

### 1.4 AABA Committees

Our Executive Committee has continued its remarkable work on behalf of the Association and its members. We continued with both routine tasks, such as award selections, and other tasks, including the revision of the *Constitution and By‐Laws*. This year, we will be joined by newly elected members, including Dr. Jonathan Bethard in the role of Treasurer, completing Dr. Graciela Cabana's term as she moves to the *Yearbook*. Dr. Bethard will be joined by newly elected Early Career Liaison, Dr. Justin Lund, and Student Liaison, Elise Adams. Please join me in congratulating and welcoming our new members. We thank Dr. Cabana as well as outgoing Early Career Liaison Katie Kinkopf and outgoing Student Liaison, Dori Kenessey. Finally, we thank Drs. Elizabeth DiGangi and Rachel Watkins for their work as Ethics Committee co‐chairs from 2021 to 2022. The Executive Committee is currently working to appoint new Ethics Committee leadership.

In addition to the outstanding work of our standing and ad hoc committees, we asked Dr. Anne Grauer to work with the Website Committee, joining Drs. Amy Rector and Ed Hagen, to consider changing our web presence. This may include changes involving the integration of membership (currently handled by a third party) with our web systems and abstract platform. The Executive Committee will be considering these matters soon.

### 1.5 AABA Awards

The most gratifying element of our annual conference is celebrating the accomplishments of our colleagues through our awards recognitions. This year, Dr. John Fleagle received the Charles R. Darwin Lifetime Achievement Award. Our Gabriel Lasker Service Distinguished Service Award goes to Dr. Andrea Taylor, and Dr. Cara Ocobock has been awarded the AABA and Leakey Foundation Communication and Outreach Award in honor of Camilla M. Smith. We are, once again, fortunate to be recognizing such a remarkable group of scholars and colleagues who continue to make fundamental contributions to our discipline.

This year's Cobb Professional Development Awards have been granted to Drs. Irisa Arney, Keegan Selic, Mayowa Titilope Adegboyega, Anna Hardin, and Amelia Villaseñor, with Honorable Mention to Mikel Arlegi. This group has crafted remarkable research projects, and the AABA is pleased to support these efforts, helping to build the foundations for our future. Please join me in congratulating these outstanding junior scholars for their work in critical areas of our field.

### 1.6 Acknowledgment and Appreciation

The AABA Executive Committee, once again, has worked diligently to serve our association and its members. Outgoing Treasurer, Dr. Graciela Cabana, has handled Association finances responsibly and carefully. Secretary Dr. Amy Rector has conducted the core business of our Association efficiently and with extraordinary care and attention. History and Honors Committee Chair, Dr. Julienne Rutherford, handled many excellent applications for our awards. Dr. Chelsey Juarez and the Student Programs Committee have been able, for the second year in a row, to support many students in traveling to the meetings through the Pollitzer Student Travel Grants. Dr. Lauren Schroeder has worked to select Cobb Grant awardees who will make a difference in our field. Dr. Stephanie Meredith helped with many new members to become a part of our Association, along with service on our HCARE committee. Early Career Liaison, Dr. Katie Kinkopf, has taken on a leadership role in addressing accessibility issues in the association. Student Liaison, Dori Kenessey, has played important roles, especially in assisting with local arrangements this year. All told, the Executive Committee has been called upon many times in the last year to advance our Association. President‐Elect, Dr. Leslea Hlusko, and Vice President, Dr. Kristi Lewton, have consistently supported our members, while contributing to the vibrancy of our annual conference and the overall health of our association. Many thanks to these colleagues for supporting the AABA in many ways.

Our journals continue to flourish through the leadership of Dr. Trudy Turner, Editor‐in‐ Chief of the *American Journal of Biological Anthropology* and Dr. Lyle Konigsberg, Editor of the *Yearbook of Biological Anthropology*. We look forward to our new *Yearbook* leaders, Drs. Sheela Athreya and Graciela Cabana, and once again, thank Lyle. We have been supported by our Wiley publisher, Gillian Greenough, and appreciate her work to support research in biological anthropology.

It is important to acknowledge and thank our business partners at Burk and Associates (Brett Burk, Lori Strong, Heide Rohland, Mary‐Lou Robinson, Shannon Graney, Jill Drupa and others). They have aided the Association in many ways, including re‐negotiating hotel contracts through the pandemic years, hosting webinars, producing monthly newsletters, and helping to undertake our multi‐format meetings. In particular, Lori Strong has been a wonderful friend for us, constantly adapting to rapidly changing circumstances and helping association leadership choose the best paths.

I want to thank our members for continuing to advance the discipline in so many ways. Hopefully, our 2023 meetings will give us opportunities to support our students and junior colleagues in advancing their important research and in setting the foundations for the future of our remarkable field.

Finally, I am delighted to have served as AABA President for the last 2 years. It has been an honor to work with my colleagues in many areas. I have been impressed by the resilience we have shown in the face of COVID‐19, even though I remain concerned about its impacts on our communities, particularly our junior colleagues. Despite these concerns, I see innovation and stunning new advances across our varied areas of research, revealing remarkable, perhaps transformative, discoveries in our future. I am grateful that the presidency has provided me with exceptional opportunities to learn more about biological anthropology and for providing me ways to engage with members in so many ways. I will continue to enjoy new findings in our field, and work to advance our research, teaching, and service in our fundamentally important discipline. Thank you.

## 2 Report of the Vice President

Kristi Lewton, the Vice President and Program Chair, presented comments and submitted the following written report:

The Vice President chairs the Program Committee and coordinates AABA programming throughout the year. In 2022–2023, this centered on organizing the 92nd Annual Meeting in Reno, Nevada and online. This is the first time that AABA has held a conference in Reno, and only the second time in Nevada. Tasks related to organizing the annual conference included program committee formation, symposium proposal review, abstract submission review and notification, workshop proposal review, webinar coordination, scheduling the conference events, publication of the annual abstract volume, and publication of the conference program (see Appendix A for the 2022–2023 timeline of these events).

As in 2022, AABA decided to hold a hybrid conference with both in‐person and virtual components for scientific presentations, workshops, and select other events. This decision was based on anecdotal reports from AABA attendees that they appreciated the opportunity to attend AABA virtually when constraints such as the COVID‐19 pandemic prevented in‐person participation.

### 2.1 Program Committee

A call for program committee volunteers was distributed to AABA members in June 2022 with a deadline to apply by July 8, 2022. Eligible regular members applied by web form. The program committee includes 69 members, a mix of returning and new members. The committee includes members from a range of career stages and international institutions. Amber Jaeger served as the assistant to the Vice President. Program committee roster for 2022–2023 was: Donovan Adams, Francisca Alves‐Cardoso, C. Eduardo Amorim, Benjamin Auerbach, Karen Baab, Shara Bailey, Heather Battles, Jonathan Bethard, Vanessa Campanacho, Tara Cepon‐Robins, Colleen Cheverko, Maria Ana Correia, Susanne Cote, Miguel Delgado, Nathaniel Dominy, Amanda Ellwanger, Nicholas Ellwanger, Kori Filipek, Rebecca George, Rebecca Gilmour, Halszka Glowacka, Jesse Goliath, Mark Grabowski, Elaine Guevara, Lauren Halenar‐Price, Angela Harden, Kevin Hatala, Amber Heard‐Booth, Megan Holmes, Jennifer Hotzman, Kent Johnson, Paige Kelmelis, Brittany Kenyon‐Flatt, Andrew Kim, Krystiana Krupa, Myra Laird, Nathan Lents, Christopher Lynn, Heli Maijanen, Lumila Paula Menéndez, Christina Nicholas, Heather Norton, Megan Perry, Marin Pilloud, Stephanie Poindexter, Sean Prall, Kathryn Reusch, Terry Ritzman, Gwen Robbins Schug, Krithivasan Sankaranarayanan, Amy Schreier, Maja Šešelj, Eric Shattuck, Michelle Singleton, Elizabeth St Clair, Katie Starkweather, Sean Tallman, Catherine Taylor, Nicole Torres Tamayo, Bethany Usher, Caroline VanSickle, Catalina Villamil, Amelia Villaseñor, Cara Wall‐Scheffler, Kerryn Warren, Julie Wieczkowski, Scott Williams, An‐Di Yim, and Chi Zhang.

### 2.2 Webinars

Demand for webinars seems to have decreased with the return to more in‐person academic events as the COVID‐19 pandemic enters its fourth year. The Program Committee solicited ideas for webinars throughout the year but had difficulties identifying individuals willing to host these events. AABA hosted two webinars in November 2022 and planned a third for early 2023 (which has been postponed). The November 2022 webinars (see below) were recorded and the recordings can be found on the AABA website.

*Understanding Genetic Ancestry and Its Implications* Co‐Sponsored by AABA and AAAG What can genetic ancestry tell us about our ancestors and disease risk? How might it influence the ways people identify themselves and communities? What are the promises and pitfalls of commercializing genetic ancestry technologies? This month, the American Association of Biological Anthropologists (AABA) is partnering with the American Association of Anthropological Genetics (AAAG) to bring a panel of three experts working in industry and academia who will discuss the broader implications of genetic ancestry for researchers, society, individual health, and identity. Join us to discuss this fascinating topic and examine the meaning and nuances of the concept of genetic ancestry.

Organizer: C. Eduardo Guerra Amorim (California State University Northridge).

Panelists:

- Graham Coop (University of California, Davis)
- Gen Wojcik (Johns Hopkins University)
- Janina Jeff (Population Geneticist and Host/Creator of *In Those Genes* Podcast)

*Discussion With the AABA Task Force for the Ethical Study of Human Remains*.

The AABA Task Force for Ethical Study of Human Remains is focused on developing a roadmap for determining what constitutes ethical study and disposition of human remains and biological samples with consent when research is warranted. This roadmap is to be based on the desires and attitudes of descendant communities and feedback from members of the AABA. The Task Force will also release a white paper with an overview of survey results from a survey focusing on how members of the AABA work with human remains. Survey data were collected in March during our conference. The white paper will be made available in advance of the webinar.

The webinar will expand on results from the survey. In addition, members of the Task Force will be available for dialogue, questions, and discussion. The webinar will provide opportunities to discuss next steps and activities for future project phases. We encourage AABA members to respond to the ideas, perspectives, and insights presented during the webinar.

Task Force Co‐Chairs: Fatimah Jackson (Howard University), Ben Auerbach (University of Tennessee, Knoxville).

Panelists:

- Jennifer Caldwell, assistant professor, Louisiana State University, Pennington Biomedical Research Center
- Carter Clinton, postdoctoral scholar, Pennsylvania State University, Department of Anthropology
- Joseph Jones, lecturer, William and Mary, Department of Anthropology
- Carmen Mosley, NAGPRA Repatriation Manager, Museum of Us

### 2.3 Invited Symposia

Proposals for invited symposia were due on August 15, 2023. The Program Committee received 13 invited symposium proposals, which were reviewed by both the Program Committee and the Executive Committee. All 13 proposals were accepted. Accepted proposals included 5 podium, 6 poster, and 2 online only (Table 1).

**TABLE 1:** Accepted invited symposia for the AABA 2023 meeting, their organizers, and format.

Invited symposia	Organizers	Format
A century of Sir Arthur Keith's stages of hominoid evolution	Scott Williams & Thomas Cody Prang	Podium
AAAG/AABA Joint Symposium: Born Which Way?: Biological and Genetic Engagement in Sex, Gender, and Sexuality Research	Christopher Schmitt & L. Zachary DuBois	Podium
American First Routes: an Interdisciplinary View	C. Eduardo Amorim & Tábita Hünemeier	Podium
Queering research: uses of LGBTQIA+ perspectives and queer theory in biological anthropology	Samantha Archer & Alexandra Kralick	Podium
Toward a holistic approach—Investigating the genetic underpinnings of phenotypic variation	Dori Kenessey & Kamar Afra	Podium
Coming to the Caribbean—Celebrating 85 Years of Rhesus macaques at Cayo Santiago; New Endeavors of Non‐Human Primate Research	Qian Wang	Poster
Forensic Anthropology as Practiced in the United States: Qualifications, Standards, and Ethical Practice	Marin Pilloud, Nicholas Passalacqua, Eric Bartelink, Melanie Beasley	Poster
Human Evolutionary Biomechanics: Past, Present, and Future	Steven Lautzenheiser & Michael Berthaume	Poster
Integrating dental wear	Almudena Estalrrich & Kristin Krueger	Poster
Symposium: A Session to Honor the Legacy of Bill Kimbel	Shara Bailey & Amy Rector	Poster
Un‐presentations: Translating Biological Anthropology Research via Creative Expression	Michelle Bezanson, Jessica Brinkworth, Negin Valizadegan	Poster
Reconstruction of activity in the past: what's going on in Latin America	Soledad Salega & Claudia Rojas‐ Sepúlveda	Virtual—Podium
Archiving and Sharing Craniofacial data: from 1D to 3D and beyond	Noriko Seguchi & Stephen Ousley	Virtual—Poster

### 2.4 Abstract Submission and Review

#### 2.4.1 Abstract Submission

The abstract submission portal was opened on September 15, 2022. Abstracts for the 2023 annual meeting were due October 17, 2022. There were a few requests for submission of late abstracts after the submission deadline, which were declined. A total of 775 abstracts were submitted, which is on par with the abstract submissions for the 2022 annual meeting in Denver, Colorado. However, these metrics still fall behind pre‐pandemic counts by 30% (e.g., there were 1121 abstract submissions in 2019).

At the time of abstract submission, authors could choose to present in‐person, online, or at both venues. Venue preferences for contributed abstracts at the time of submission were: in‐person 80%, online 9%, both 11%. However, in the months leading up to the conference, there were several requests to change from online to in‐person or from both to in‐person only. In other words, as the conference neared, there was a trend toward attendees changing their venue preference to in‐person only. Venue preferences for contributed abstracts as of April 3, 2023 are: in‐person 84%, online 8%, both 7%.

#### 2.4.2 Abstract Review

Abstracts were assigned to two members of the Program Committee for review with a review deadline of November 18, 2022. Most reviews were received by the deadline, and all reviews were received by November 23, 2022. Abstracts with one or more recommendations to reject received an additional round of review. After this second round of review a total of 11 abstracts had received at least two recommendations to reject. I evaluated each of these, offered revision based on reviewer comments, and ultimately rejected 2 abstracts. Abstracts were rejected on the basis of a lack of evidence of data analysis or results. Authors of rejected abstracts were notified on December 1, 2022.

Authors of accepted abstracts were notified on December 5, 2022. Following the advance team visit to Reno, Nevada on February 1–3, 2023, authors of accepted abstracts were notified of the presentation schedule on February 10, 2023.

### 2.5 Workshop Proposals

We received 11 proposals for workshops by the November 15, 2022 deadline. Workshop proposals were evaluated by AABA President Steve Leigh and me. All workshop proposals were accepted (Table 2) and organizers were notified on December 17, 2022. Two workshops were later withdrawn by the organizers. Three additional workshop/panel events were planned by AABA committees. Three of these events are scheduled to be live streamed to the virtual platform.

**TABLE 2:** Accepted workshops for the 2023 AABA meetings, the dates of their presentation, and their organizers.

Date	Workshop/panel	Organizers
19‐April	SlicerMorph: an open‐source toolkit for 3D imaging and morphometric analysis^a^	Chi Zhang & Murat Maga
19‐April	Introduction to Ancient DNA Analysis^a^	Elena I. Zavala, Ainash Childebayeva, Laurits Skov
19‐April	Up Goer Five: Using Simple Language to Communicate Your Research to the Public	C. Kinley Russell & Briana Pobiner
19‐April	COD‐WIN Panel: Experiencing and negotiating the dynamics of power: beginning the conversation	Anne Stone & Michelle Bezanson
20‐April	Social Network Analysis in R for Biological Anthropologists	James Holland Jones
20‐April	Primate Interest Group Workshop	Lydia E. O. Light, Michelle Rodrigues, Andrew Halloran
20‐April	Professional Development Committee Workshop: Let's Get Funded! Strategies for Writing Successful Grant Proposals^a^	Lauren Schroeder
20‐April	Improv for Anthropologists: Boosting creativity, confidence, and collaboration for teachers, researchers, and public outreach through improvisational training	Amanda L. Ellwanger, Marc Kissel, Natalia Reagan
21‐April	Training for Equitable Peer Review in Biological Anthropology	Trudy Turner & Sheela Athreya
21‐April	Leadership in a liminal space: chairing departments during times of uncertainty	Amy Rector
21‐April	Identifying and Recording Periosteal New Bone Formation (PNBF) in Fetal‐Infant Individuals	Claire Hodson
22‐April	COD Disability workshop: Dialogues and Pathways Toward a more Accessible Biological Anthropology	Katie Kinkopf & Saige Kelmelis

^a^ Indicates workshop was recorded and live streamed.

### 2.6 Scientific Session Formation and Advance Team Visit

Scientific sessions were assembled by seven subcommittees of the Program Committee (Bioarchaeology, Forensics & Dental, Functional Anatomy, Genetics & Education, Human Behavior & Biology, Primatology, and Paleoanthropology). Each subcommittee had 3–4 members (i.e., 27 members of the Program Committee were on subcommittees). Each subcommittee was tasked with grouping contributed abstracts into podium, poster, and virtual sessions and assigning chairs to each session. Subcommittees were given a month to complete this task, and all sessions were formed by mid‐ January.

The number of podium sessions per subject area was based on the proportion of abstracts in each subject area:

- Bioarchaeology = 4
- Dental anthropology = 1
- Education in biological anthropology = half session
- Forensic anthropology = 1
- Functional anatomy/tissue biology = 2
- Genetics and genomics = 2
- Human behavior = half session
- Human biology = 2
- Paleoanthropology = 3
- Primatology = 3

The AABA Advance Team visited the conference venue in Reno from February 1–3, 2023. The team included Lori Strong (Burk and Associates), Jonathan Bethard, Leslea J. Hlusko, Steven R. Leigh, Stephanie Meredith, Marin Pilloud, and Richard Scott. Because the sessions were formed prior to the advance visit, the team focused on scheduling sessions (assigning sessions to rooms, days, and times) and entering the sessions into the AABA meetings database.

The programming schedule during the virtual week of the conference was based on presenters' individual time zones. Presenters in the virtual conference were located across the globe, with presenters in, for example, Japan, Australia, South Africa, Argentina, and California. A survey was sent to all presenters asking them to indicate their availability on April 3–5 and session times were based on survey results. As a result, the live, virtual sessions ran from 2 a.m. to 7:30 p.m. PDT. BAI staff ran the AV for the virtual sessions (i.e., administering the Zoom meeting and livestreaming the session to the X‐CD platform) with the exception of three virtual sessions that convened outside of EDT business hours, which were staffed by Kristi Lewton and Jonathan Bethard.

### 2.7 Abstracts

**FIGURE 1:**
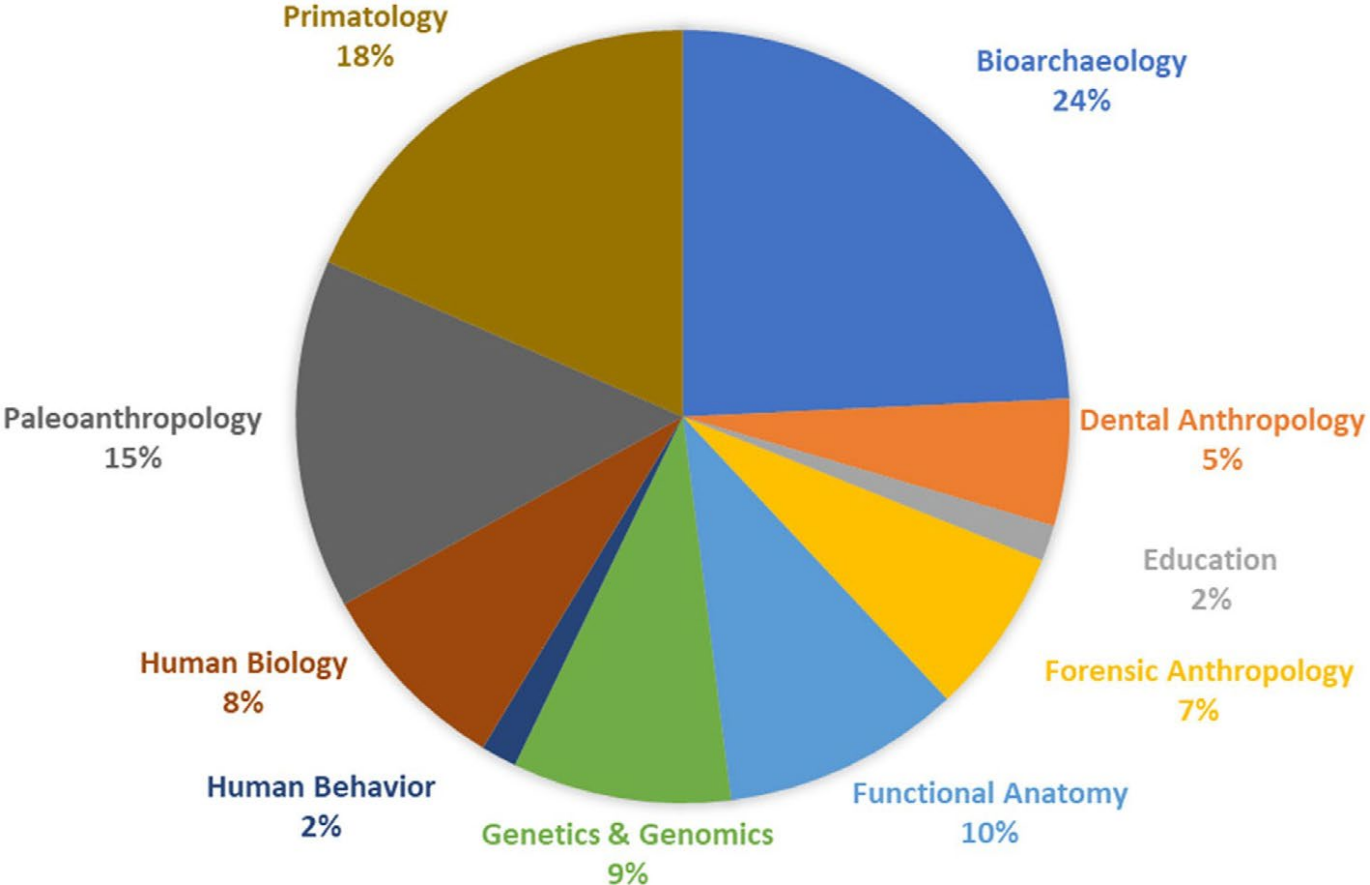
Proportions of contributed abstracts in each of the 10 major topic areas.

As of April 3, 2023, there are 602 contributed abstracts and 154 invited abstracts for a total of 756 abstracts. The proportion of contributed abstracts by subfield is in Table 3 and Figure 1, and historical data concerning abstract submissions and other meeting metrics are available in Appendix B. The abstract volume was submitted to Wiley on February 16, 2023 and proofs were submitted on March 22, 2023.

**TABLE 3:** Number of contributed abstracts in each of the ten major topic areas.

Topic area	Number of abstracts
Bioarchaeology	146
Dental Anthropology	32
Education	9
Forensic Anthropology	42
Functional Anatomy	60
Genetics & Genomics	55
Human Behavior	9
Human Biology	50
Paleoanthropology	88
Primatology	111

### 2.8 Meetings Program, Virtual Platform, and Accessibility

AABA signed a contract with a new virtual platform in February 2023–X‐CD– after the previous virtual platform that was used in the 2022 and 2021 conferences (Pathable) was discontinued. Materials uploaded to the platform (i.e., posters and videos) and recorded sessions will be viewable through September for registered conference attendees.

The meetings program was finalized by February 10, when authors were notified of their presentation schedule. The first draft of the full meetings program was published on the AABA website on March 1. Updates were published thereafter until the program was sent to the printer on March 31, 2023.

Live attendance at virtual sessions ranged from 18 to 48 individuals (including AABA/BAI staff attendance).

To increase accessibility of the annual AABA conference, we created a survey that accompanied abstract submission to query attendees on accommodation needs (e.g., oral presentation transcription, single level/no stairs). This effort was led by Early Career Liaison Katherine Kinkopf. Of the surveys submitted by the abstract submission deadline, 2% requested an accommodation.

All podium presentations use voice‐to‐text transcription using PowerPoint subtitles. We have a quiet room with low lighting and reduced sensory stimulation, a Family and Lactation Respite Room, and access to a secure refrigerator for medications. We also have proximity badge stickers for attendees to indicate their preference for physical space while interacting with colleagues.

### 2.9 Meeting Registration

As of April 17, there are a total of 1306 individuals registered for the 2023 annual meeting. International registrants from 37 countries make up 18% of meeting registrations, which is on par with meetings back to 2019 (Appendix C).

### 2.10 Acknowledgments and Thanks

I sincerely thank everyone who helped organize the 2023 program. I am immensely grateful to the 69 members of our Program Committee who conducted and completed symposium and abstract reviews thoughtfully, thoroughly, and expeditiously. Additional thanks go to the 27 members of the Program Committee who took on the task of organizing the contributed sessions for both the Reno and the Online programs.

Thank you to the Advance Team who met in Reno to set the schedule for the Reno program: Jonathan Bethard, Leslea J. Hlusko, Steven R. Leigh, Stephanie Meredith, Marin Pilloud, and Richard Scott and to the graduate student volunteers Ashley Baeza, Reecie Dern, Dori Kenessey, and Emily Smith.

Thank you to Ed Hagen, our website and abstract database developer, without whom our program would not be possible. The Program Assistant Amber Jaeger has been a tremendous help and I am very grateful for her amazing organizational skills. I also want to thank Maggie Hoffman for lending her incredible primate images to the AABA meetings' swag.

I thank our partners from Burk & Associates, including Annual Meeting Executive Director Lori Strong, Heide Rohland, Mary Lou Scarborough, Brett Burk, Cooky Bysura, Jill Drupa, Amy Sullivan, Sean Sullivan, Raelene Sok, Elizabeth Terry‐Humen, and Ruedi Birenheide. This team helped with registrations, organizing student volunteers, sending emails to the membership, producing our printed materials, interfacing with the exhibitors, and coordinating webinars.

Thank you to our hosts in Reno, Marin Pilloud and Richard Scott, for helping to make this meeting a success.

## 3 Report of the Secretary

The following report was submitted by Amy Rector:

### 3.1 Communications and Social Media

Communicating opportunities and events with AABA membership is a primary responsibility of the Secretary. For 2022–2023, our primary avenues of communication have included the website, email blasts through BAI, and AABA Twitter and Facebook. We often receive feedback that our emails are spammed, and on both social media platforms engagement from membership has felt relatively low. We have 7570 Twitter followers, and our last 28 days have included an increase of tweet numbers (88.9%), tweet impressions (40 K; up 71.0%), profile visits (2268; up 50.3%), mentions (77; up 75.0%), and followers. For the same time period on Facebook (with the same posts), posts reached a total of 6935 people and were engaged with 413 times. In an effort to drive up engagement, I have started creating imagery for events, invited symposia, workshops, and others to share on Twitter and Facebook.

Anecdotally, this strategy might be improving our visibility.

Two webinars were posted to the YouTube page (see the VP's report for their descriptions) and we will launch the AABA Oral History Project shortly after we conclude this year's meeting (see the History and Honors report for more information).

### 3.2 AABA Webpage

An ad hoc committee was formed by the President to begin investigating a potential transition from the current AABA webpage to a new platform. This committee is chaired by Anne Grauer, and includes the Secretary and Webmaster Ed Hagen. The current website is a bespoke system that is both our primary forward‐facing platform for communicating with membership and the public, and our record keeping system for meetings and proceedings. Goals of transitioning to one or more new platforms include (1) integration of all record keeping needs (including membership management) and forward‐facing communications, and (2) functionality and flexibility as our needs change. Some of our primary concerns as a committee include (1) one‐time and annual costs associated with a website build and maintenance, (2) transition and maintenance of our stored content, and (3) ability to tweak platforms for our specific needs. We have met with several members of BAI and representatives from other organizations with similar membership and needs to discuss experiences with potential platforms, and are currently in the process of vetting potential options.

## 4 Report of the Treasurer

The following report was submitted by Graciela Cabana for the fiscal year 2022:

This report from the Treasurer represents a preliminary account and assessment of the AABA finances between January 1st and December 31st, 2022. This report is preliminary because the accounting books for the 2022 fiscal year do not officially close until August 2023.^1^ By this point, however, most income and expenses for 2022 have been recorded.

At the close of 2022, the AABA showed a net operating cash loss of **$120,912.78** due to the normal expense of holding an in‐person annual meeting (Table 4). The AABA's total cash‐on‐hand for operational purposes was **$491,232.12** at the end of 2022; this amount includes our bank balances that include a 3.5% infusion from our investment income^2^ (Table 5).

**TABLE 4:** Operating gains/losses over 6 years.

	2017	2018	2019	2020^a^	2021^b^	2022
Income	$678,258.40	$648,313.02	$708,058	$419,810	$340,154	$421,281.09
Expenditures	$710,948.30	$899,049.55	$837,651	$401,412	$289,965.85	$542,193.87
TOTAL	($32,689.90)	($250,736.53)	($129,593)	$18,398	$50,188.15	($120,912.78)

^a^ Annual Meetings canceled due to the COVID‐19 pandemic.

^b^ Annual Meetings held virtually due to the ongoing COVID‐19 pandemic.

**TABLE 5:** Cash‐on‐hand over 2 years.

	12/31/2021^a^	12/31/2022
TOTAL	$415,813.27	$491,232.12

^a^ Annual meetings held virtually due to the ongoing COVID‐19 pandemic.

### 4.1 Income

The AABA's 2022 income derived from several sources, primarily membership dues ($154,010), annual meeting registrations ($179,379), the auction ($7954), exhibitors ($5275), and donations ($5213). Our 2022 income also included the annual sponsorship grant from Wiley for $55,000. After 2019 and coinciding with the COVID‐19 pandemic, membership numbers decreased precipitously (see the Membership Chair's Report), likely because membership renewals are tied to annual meeting registrations. The resulting financial blow to our operating costs has been somewhat mitigated by the fact that since 2020, dues for regular members are now charged at a ~30% higher rate (from $130 to $170 annually).^3^

### 4.2 Expenditures

Major 2022 expenses included costs related to the Annual Meeting such as A/V, catering, invited speakers, the meeting app, etc. ($284,020.82) and fees for membership, meeting, and accounting services provided by Burk & Associates and Gelman, Rosenberg, and Freeman ($138,064.71). Accountant services include our annual review and tax filing (available to any member by written request to the Treasurer), and bookkeeping. Other expenses ($42,883.69) include credit card fees, Executive Committee expenses (including VP support, travel, and supplies), and legal fees to Allison, Slutsky and Kennedy. In calendar year 2022, the AABA also dispersed $58,096.35 in awards, including Cobb Professional Development Awards to support early career member research and Student Presentation awards to support student member research.

### 4.3 Investments

The AABA's long‐term investments, managed by Merrill Lynch, suffered some loss in 2022. In 2022, our portfolio declined by ~20%: the net portfolio value at year‐end 2021 was $4,776,795, declining to $3,803,745 by December 31, 2022. Investment funds are allocated between equities (in 2022 about 80% of our assets), fixed income (comprising 19% of our assets), and 1% cash. As always, the AABA works with our financial consultants to evaluate the allocation of funds to ensure that the proportion of funds in equity, fixed‐income accounts, and cash reflects the needs and goals of the AABA.

The AABA transfers 3.5% of its net investment portfolio (5‐year average) to our operating accounts on an annual basis. With this annual infusion into our operating budget, the AABA supports awards, grants, and programming during the annual meetings. In 2022 the amount transferred was **$134,844.37**.

### 4.4 Summary

We will need to remain watchful of our annual finances given that every year since 2019 has brought new challenges, starting with the COVID‐19 pandemic, and currently, the U.S. and global economies. At this point, however, the AABA remains a financially healthy association, and we expect to continue developing new initiatives and offering valuable programming for our members.

### 4.5 Acknowledgements

Thank you to Laurie Mullins (Burk and Associates) and Rob Clayton (Clayton and Associates) for their high‐ level support during my time as AABA Treasurer.

## 5 Report of the Editor‐In‐Chief: *American Journal of Biological Anthropology*

The following report was submitted by Trudy Turner:

### 5.1 General Remarks

While this year has been a more typical year for the journal, we are still seeing some lingering effects of the global pandemic. As with most Wiley journals, the number of published manuscripts is down somewhat; we are also seeing an increase in time from submission to acceptance for articles. These seem to be industry‐wide trends. Everyone associated with *AJBA* remains committed to providing the best possible science in a timely manner.

The journal has instituted several changes this year. Probably the most notable has been the separation of the *Yearbook* and *AJBA* web presence. In terms of the review process, we instituted coordination of decision terms. In the past, editorial board members and associate editors had a different list of potential decisions than the editor‐in‐chief. These are now coordinated and everyone is using the same decision terms. We have also shortened the time to decision for all members of the editorial board. We hope this will facilitate the timely handling of manuscripts. We have also instituted a policy that we will not publish a manuscript until the Data Availability Statement is complete and easily found in the manuscript. We have had a requirement for a data availability statement for 2 years, but now will no longer issue a final acceptance of a manuscript without the statement being clearly evident. Last year we instituted a requirement that authors of manuscripts that dealt with human genetics or human remains fill out a form entitled Genetics Research of Human Remains and provide a blank informed consent form to be included in Supplemental Materials not for Review. The material on the form would also be summarized in the Materials and Methods section of the manuscript. The requirement has been in place for a year and the editorial board is currently engaged in reviewing how this requirement has been working. We always understood that the requirement would be reviewed and we welcome any comments that members of our community wish to provide.

If we feel a manuscript submitted to *AJBA* is better suited to another journal, we have the ability to either transfer or refer the manuscript. If the manuscript is clearly out of the scope of the journal, I can immediately transfer it to a Wiley service that will try to place the manuscript appropriately. If the manuscript is potentially of interest to the readers of the journal, I will confer with an Associate Editor and together we will make a decision about either having the manuscript reviewed or referring it to a journal with which we have a cascading arrangement. We can refer manuscripts to the *Yearbook of Biological Anthropology*, the *American Journal of Human Biology*, the *American Journal of Primatology*, the I*nternational Journal of Osteoarchaeology*, the *Journal of Forensic Science*, and the *Anatomical Record*. These journals can also refer manuscripts to the *AJBA*. We are currently exploring other journals to add to our cascade list. In 2022, 11% of articles submitted for publication were transferred and 9% were referred to journals in our cascade. Approximately 48% of both transferred and referred articles were submitted to other journals and about 20% of these were published elsewhere; of the total articles sent out, 10% were ultimately published.

This year we will offer authors the opportunity to provide a second or third language abstract. Authors will be responsible for the translations and these will not be copyedited by the production staff. We hope all authors will take advantage of this and make their work accessible to a wider audience.

Although we did not publish any special issues this past year, we have seven in process; three are very near completion and will be published this year. We welcome additional ideas for special issues. Please feel free to contact me if you have an idea.

The 2022 editorial board included seven associate editors, 32 editorial board members, seven early career editorial board members, a digital fellow and two media review editors. Several members of the editorial board are leaving the board this year. I am incredibly grateful to Sheela Athreya, Hallie Buckley, Omer Gokcumen, Karim Quattara, Laurie Reitsema, Amanda Thompson, Tiffany Tung and Laura Wilson for their exemplary service on the board. Being a member of the editorial board requires time and dedication and all our members have worked very hard. I am also indebted to Kieran McNulty for his service as associate editor. I am incredibly grateful to Kieran for his service. I am also especially grateful to Graciela Cabana and Lauren Schroeder for their editorship of Media Reviews. They are also leaving the journal this year. Thank you to all editorial board members for your efforts. Mark Kissel and Ryan Harrod have taken over as editors of the reviews and we all look forward to your continuing the exceptional work of Graciela and Lauren. This year we welcomed Kanya Godde Chrisco, Noreen von Cramon‐Taubadel, and Jamie Hodgkins to the editorial board. We will be adding additional members during the year. We are accepting applications and recommendations for these positions. We have publicized this call on the AABA website and through social media. If you are interested, please contact me.

At the meeting this year, Sheela Athreya and I offered a workshop on Equitable Peer Review in Biological Anthropology. We hope to offer this workshop every other year and offer other workshops in the future.

Being somewhat over halfway through my tenure as Editor‐in‐Chief of the journal prompted me to assess my goals for the rest of my time with the journal. I plan to work on two areas‐ increasing the percentage of open access publications in the journal and strengthening and supporting the journal's relationship with the association. Currently about one‐third of the articles we publish are open access. My goal is to try to increase that by % a year, so that in 3 years we will be at close to two‐thirds open access. One way to do this is to take better advantage of Wiley's transitional deals or transformational agreements. These are contracts between institutions (libraries, national or regional consortia) and publishers that transition the business model over time from one based on a paywall to one where publishers are paid for open access publishing services. At this point Wiley has 65 of these contracts; most include multiple institutions. This is nearly double the number of contracts that were available last year. We hope to offer webinars to authors in countries that have these agreements about the scope and aims of the journal and further encourage appropriate submissions. In order to support the relationship between the journal and the association, we are beginning discussions on ways to recognize reviewers to the journal. I hope to be able to report on this further next year and in editorials in the journal.

### 5.2 Metrics

**FIGURE 2:**
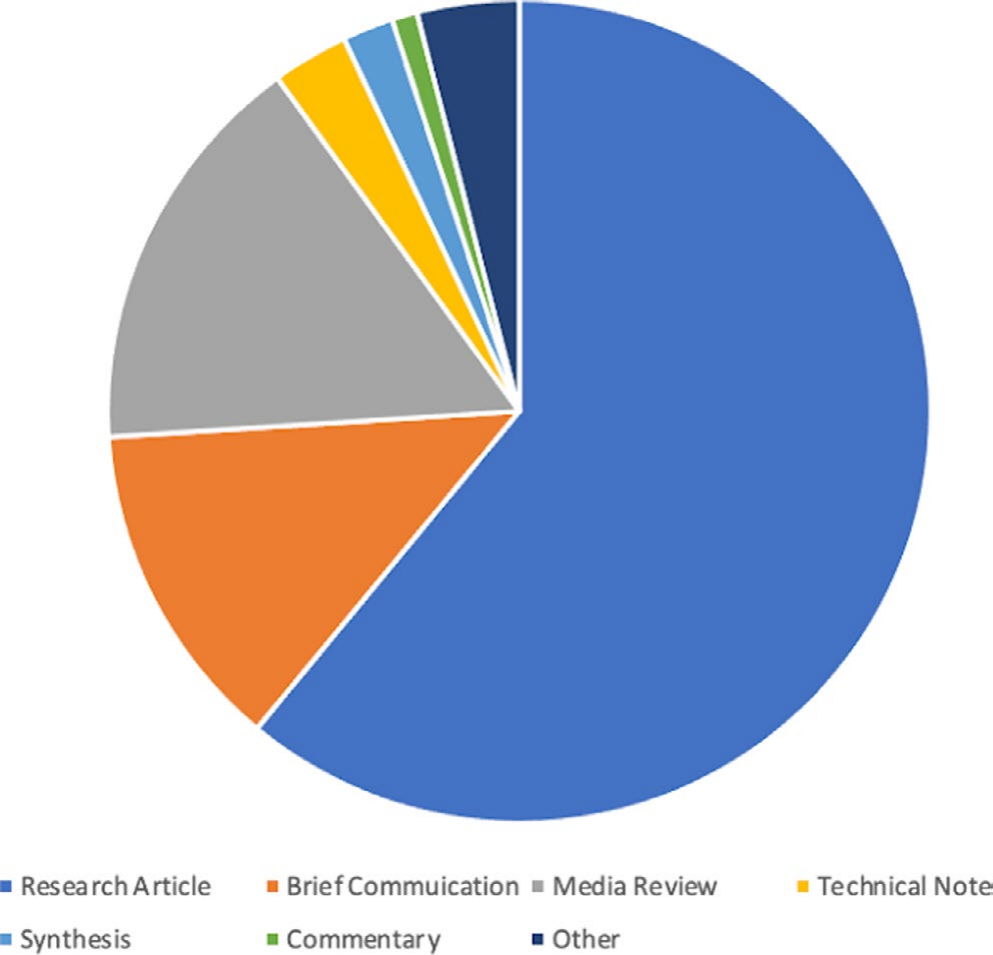
Proportions of articles by type submitted to the American Journal of Biological Anthropology.

During 2022, the journal published three volumes (177–179), each of which had four issues with a total page count of 2185. This total is less than 2021, but is closer to what we have published in previous years. This does not include the two supplements: the annual meetings issue and the *Yearbook of Biological Anthropology*. In 2022, we received 382 submissions and published 186 manuscripts. Submissions to the journal increased slightly over 2021 (1.1%) while published content decreased by 9.7%. The published content included: research articles (61% of the total published pages), brief communication (13%), media reviews (16%), technical notes (3%), synthesis (2%), commentary (1%) and other (including resources, review articles, letters to the editor, editorials—all at less than 1%) (Figure 2).

Although we received 382 original submissions, this number does not represent the number of requests we send out for review. Almost every article is revised and resubmitted, often more than once. We send out over 1300 requests for review. We are incredibly grateful to everyone who reviews for us; however, nearly half of all review requests are declined. Unfortunately, this has led to an increase in the time from submission to acceptance or rejection. Although our median for decision is 49 days, this includes all transfers and referrals. This number increases substantially if those articles are removed. Please accept our gratitude for the work you do and our encouragement to continue to do so.

The acceptance rate for 2022 for all material was 49%. Articles submitted from the United States constitute 37% of all articles. The countries with the next greatest number of submissions following the US are China, the United Kingdom, Spain, Canada, India, France, Australia, Poland, Brazil and Argentina. We continue to reduce the number of days from receipt of a final manuscript to Early View, which is to the credit of the Wiley production staff.

Last year there were approximately 748,000 downloads of articles, down about 20% from the year before. Citations of articles in the journal are steady at about 100,000 a year. The impact factor calculated by the ISI/Web of Science for 2021 was 2.963. The *AJBA* ranks 15/93 in Anthropology journals and 30/50 in Evolutionary Biology journals. Although I will continue to report IF for the journal, I would like to call your attention to the DORA agreement recently endorsed by Wiley. The San Francisco Declaration of Research Assessment's overarching goal is to shift emphasis away from journal‐based metrics, toward article‐level metrics and individual author contribution for a broader, more equitable view and assessment of research impact. In order to accomplish this, journals will greatly reduce emphasis on the journal impact factor (IF) as a promotional tool, make available a range of article‐level metrics, and encourage responsible authorship practices and highlight the specific contributions of each author. You will notice in the future additional metrics on the *AJBA* homepage including the Journal Citation indicator.

**FIGURE 3:**
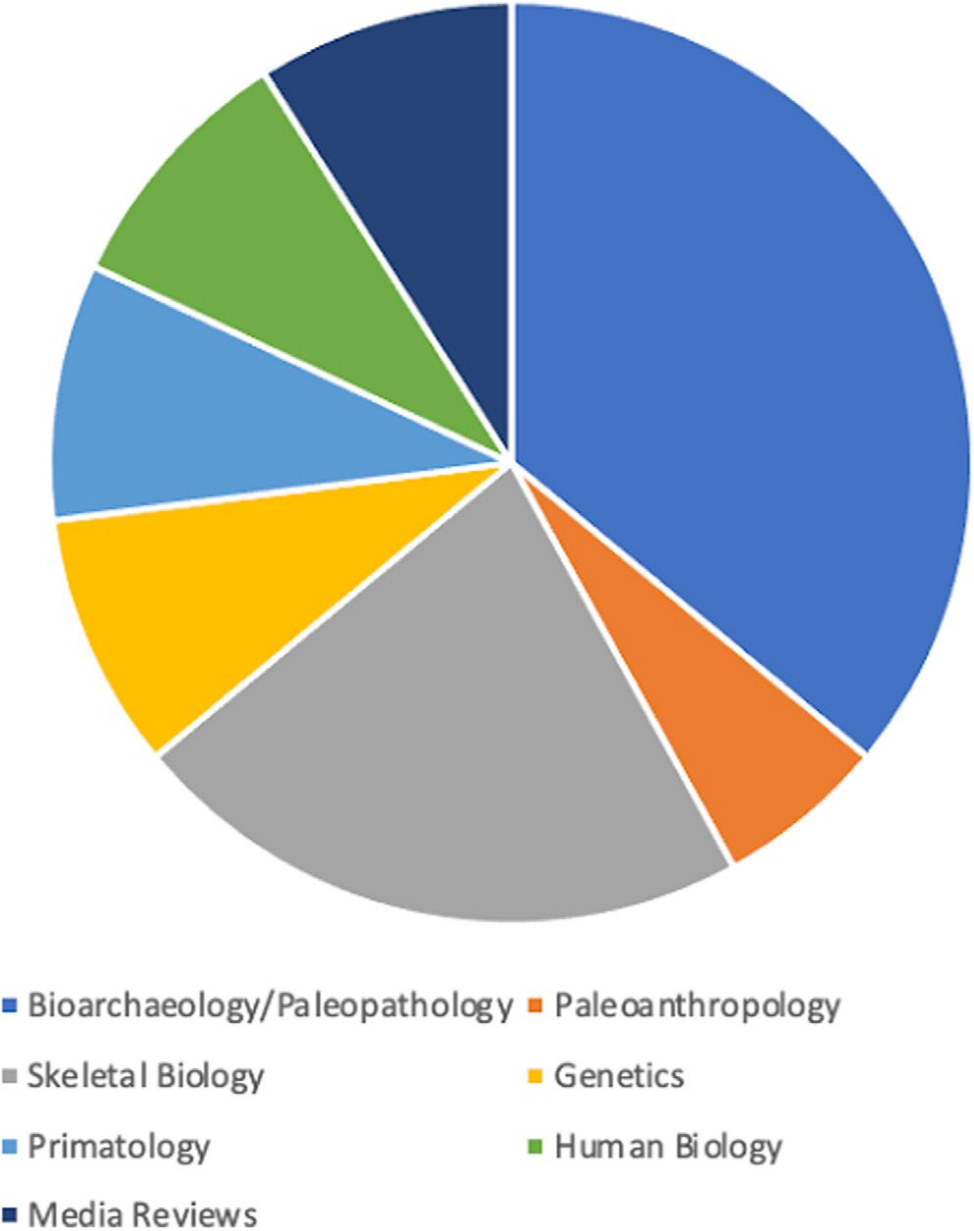
Proportions of articles by topical area published in the American Journal of Biological Anthropology.

Research publications can be broken down into six general subject areas: Bioarchaeology/Paleopathology (36%), Paleoanthropology (6%), Skeletal Biology (22%), Genetics/Genomics (9%), Primatology (9%), Human Biology (9%), Media Reviews (9%) and Other (1%) (Figure 3). One of my goals is to have every issue of the journal include articles of interest to all the membership. In order to achieve this, I encourage all members of the association to send their best work to the *AJBA*.

### 5.3 Acknowledgments

I am extremely grateful to the Associate Editors and the Editorial Board Members of the journal. I remain in awe of your dedication, insight, care and diligence as you shepherd manuscripts through the process. Your work has been remarkable. For those retiring from the editorial board this year—again thank you for your dedication and your time. I am also extremely grateful to all the reviewers. It is your work that allows us to continue our work. The peer review process depends on reviewers who take their time to ensure the best possible science. Reviewers give us their time when they are undoubtedly pulled in many, many different directions. You are the heart of this process and all of us are so grateful for your efforts. I am also very grateful to Gillian Greenough, our publisher at Wiley. Gillian has been a great partner in navigating some crucial issues the journal has faced. Gillian and the staff, especially Felicia Bonanno at Wiley, have been vital to the continued success of the journal.

Thank you also to Reeni Sunder and the production staff for their attention to so many details and their excellent work. I am deeply grateful to the AABA for the opportunity to edit the journal. Thank you all.

## 6 Report of the Outgoing Editor‐In‐Chief: *Yearbook of Biological Anthropology*

The following report was submitted by Lyle Konigsberg:

The 2023 edition of the *Yearbook* is my last (fifth) as Editor. I would first like to offer my sincere thanks to Jada Benn Torres, Jane Buikstra, Noreen von Cramon‐Taubadel, Darryl De Ruiter, Eduardo Fernandez‐Duque, Agustín Fuentes, Debbie Guatelli‐Steinberg, and Laura MacLatchy who have served as the editorial board.

As of this writing, six 2023 *Yearbook* articles are in Early View and one more is in production with Wiley. The titles and authors are:

- Morphology, evolution, and the whole organism imperative: why evolutionary questions need multi‐trait evolutionary quantitative genetics (Auerbach et al.)
- The energetics of childhood: Current knowledge and insights into human variation, evolution, and health (Urlacher)
- Evolutionary biological perspectives on current social issues of breastfeeding and weaning (Tsutaya and Mizushima)
- Genomic structural variation: A complex but important driver of human evolution (Soto et al., Dennis as corresponding author)
- African apes and the evolutionary history of orthogrady and bipedalism (Williams et al.)
- Form, function and evolution of the human hand (Kivell et al.)
- Integrating genealogy and dental variation: contributions to biological anthropology (Paul et al.)

My Preface to the volume is nearly complete and the cover is done. As soon as the final article is in Early View, I can release the 2023 *Yearbook*. With any luck, this will occur before our meetings in Reno, where I will pass the reins to Graciela Cabana and Sheela Athreya as the new co‐editors. I look forward to reading the next five *Yearbooks* released under their highly capable guidance. Be sure to visit the webpage at: https://www.yearbookbioanth.com/ to see more about the upcoming 2024–2028 *Yearbook*s.

**FIGURE 4:**
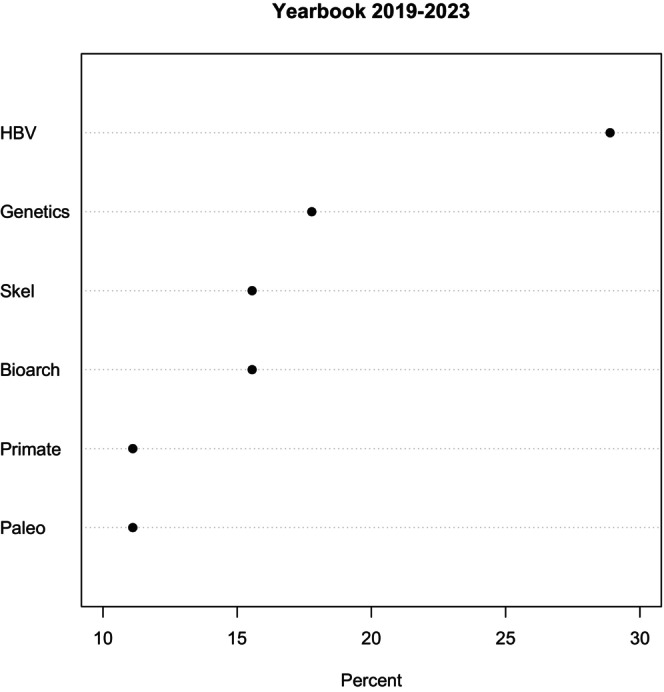
Percent of submissions to the Yearbook from 2019 to 2023 according to topic.

It has become a tradition with the final *Yearbook* report of an Editor‐in‐Chief to provide some information about the past five volumes. There were 33 articles published in the five volumes. The traditional classification for articles is genetics, paleo, primate, skel/bioarch, HBV (for human biological variation), and other (Figure 4). I have split “skel” from “bioarch” and found that, as one would expect from *Yearbook* articles, most can be classified into two or even three categories. I have done away with “other” as there was only one article that (sort of) fit that category, primarily because it could be counted in every category.

I began by thanking the editorial board and I would like to close by thanking Gillian Greenough, Reeni Sunder, and Olivia Barry from Wiley for their instruction on basic editorial issues and the use of “Scholar One.” They, together with the editorial board, have made the job of editor that much easier as well as enjoyable. And if somewhere along this five‐year path I exposed my snarlier side (my evil twin “Kyle”) I do apologize.

## 7 Committee Reports

The AABA committee chairs provided written reports in electronic form in advance of the meeting (reports appear below), and the floor was opened for questions.

The Membership Committee report was presented to attendees in electronic form online, and orally at the Business Meeting by Stephanie Meredith. President Steve Leigh called for objections to new members. In the absence of objections by voting members in attendance, Membership Committee Chair Stephanie Meredith cast a unanimous ballot for the list submitted.

### 7.1 Membership Committee

Stephanie Meredith submitted the following written report on March 15, 2023:

### 7.2 Membership Trends 2013–2023

Membership numbers and % yearly change reflect end‐of‐year totals, except for 2023 (Table 6). At the time of the 2022 AABA Member Report (3‐23‐2022), there were a total of 1448 members. This year's membership count (as of 3‐15‐2023) is shy of last year's pre‐meeting numbers by 73, indicating a decrease of 5% compared to a similar time last year. It is customary to report this metric. However, since 2017, the difference in total memberships from the time of the pre‐meeting data presentation to the end of the year has ranged from 80 (in 2020) to 574 (in 2018), and year‐to‐year differences in pre‐meeting membership numbers are more variable than year‐to‐year differences in end‐of‐year membership numbers. Therefore, it is unclear whether a 5% decline in pre‐meeting membership numbers has any predictive value with respect to total membership trends. Student member numbers held steady since the end of membership year 2022, but regular member numbers have decreased, suggesting that regular members are not renewing regularly. Low membership totals may justify more active outreach attempts aimed at building back our membership.

**TABLE 6:** Membership trends: 2013–2023.

2013	2014	2015	2016	2017	2018	2019	2020	2021	2022	3/15/2023
1457	1563	1950	2271	2263	2254	2074	1963	1474	1558	1375
% change	+7.3%	+24.8%	+16.5%	−0.4%	−0.4%	−8.0%	−5.4%	−24.9%	+5.7%	

### 7.3 Membership Demographics 2022

**FIGURE 5:**
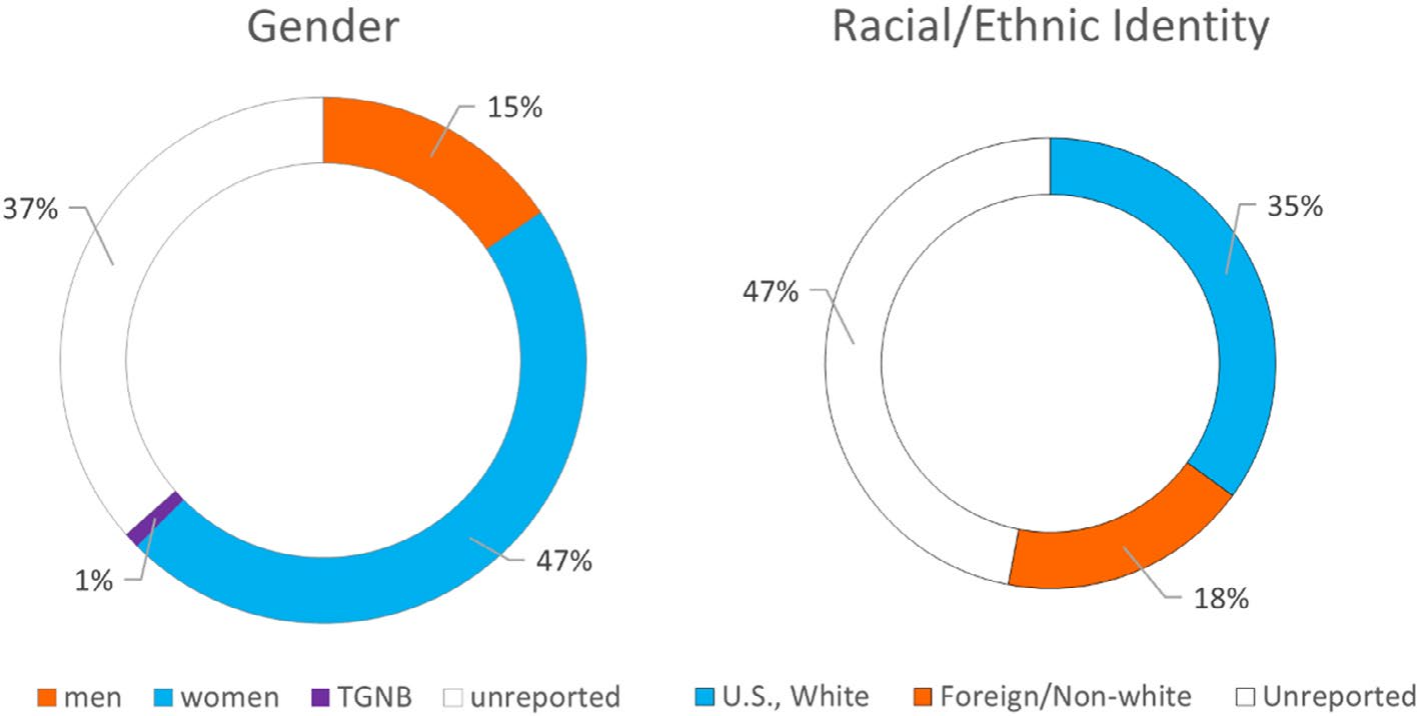
Some available demographic information about AABA membership. TGNB, transgender/non‐binary.

I would like to be able to report on membership demographics, as the association has an interest in understanding demographic changes in its membership through time. However, we do not possess these data for a large proportion of our membership (but see Figure 5 for a sample). Changes to the membership renewal process to facilitate gathering these data upon membership renewal may be warranted.

### 7.4 Membership Applicants 2022–2023

Between March 20, 2022 and March 15, 2023, a total of 335 people newly applied for AABA Membership and were provisionally approved as new members (Table 7). (New membership applications prior to the 2022 meetings numbered 337.) Of student applications, 67% were from graduate students and 33% were from undergraduates. All new applications require approval by the membership. Seventy‐eight applications from existing members were provisionally approved to transition to new membership categories (Table 8). Some of these transitions do not require a membership vote unless the applicants' membership has lapsed for 5 years or more. The names of members whose membership changes do not require membership approval are excluded from the list of names to be voted on.

**TABLE 7:** Applications for new membership.

Membership category	#	% of new memberships
Regular/postdoc/contingent (voting members)	72	21.5%
Special	12	3.5%
Student	251	75%
Total new members	335	

**TABLE 8:** Applications for changes in membership status.

Previous member category	New member category	#
Postdoc/contingent	Regular	15
Regular	Retired	10
Regular	Postdoc/contingent	3
Special	Regular	2
Student	Postdoc/contingent	22
Student	Regular	18
Student	Special	4
Special	Student	3
Special	Postdoc/contingent	1

New and change applications continue to come in. Names of applicants who have been provisionally approved but require membership approval have been added to the list of names to be voted on at the annual business meeting up (Appendix D) until April 14, 2023, but their applications are not reflected in the summaries that have been presented above. Names of applicants who are provisionally approved after April 14, 2023 but require membership approval will be included in the membership report for 2024.

### 7.5 Professional Development Committee

Submitted by Lauren Schroeder.

### 7.6 Early Career Liaison Program

Dr. Justin Lund (Postdoctoral Inclusive Academic Excellence Scholar, Northern Arizona University) was chosen as our new Early Career Liaison. In his application, Dr. Lund proposes the creation of a Committee on Diversity (COD) interest group dedicated to Indigenous biological anthropologists as a networking, activist, and intellectual space; a unified Indigenous presence supported by the AABA. This proposal stems from the recognition by Dr. Lund and other Indigenous colleagues that although the practice of anthropology is now a more collaborative endeavor between community and scholar, the legacy of colonial practice still permeates our association in various ways. Dr. Lund will be working closely with the Chair of the COD, Susan Antón, to reach these goals, and to identify a set of guidelines to make anthropological spaces more welcoming to Indigenous peoples and students. We wish him the best during his appointment as the AABA Early Career Liaison, and we thank Dr. Katherine Kinkopf for her service as outgoing Early Career Liaison.

### 7.7 Cobb Professional Development Grants (CPDG)

The AABA CPDG committee reviewed 28 eligible applications for the 2022–23 grant cycle. The applicants spanned multiple continents, with all subfields of biological anthropology represented. Overall, the pool of applications was very strong, reflecting the excellence of our early career investigators. Each application was reviewed by three committee members. In reviewing proposals, the committee considered the significance of the work, the quality of the proposal, the potential impact of the award on the applicant's career (including career stage and access to other funding sources), and the strength of the recommendation letter and CV. The committee recommended funding five proposals, up to $7500 each, and one honorable mention. Award and declination letters were emailed to applicants on March 17, 2023. The award recipients are as follows:

**Mayowa Titilope Adegboyega**.

Postdoctoral Research Associate, Duke University.

*Improving Hemipelvectomy Patient Outcomes using Evolutionary Concepts of Hominin Locomotion*.

**Irisa Arney**.

Assistant Professor, Western University of Health Sciences.

*Middle Miocene environments from herbivore stable isotopes and assessing C4 biomass seasonal variability*.

**Anna Hardin**.

Assistant Professor, Western University of Health Sciences.

*Evaluating evolutionary change in tooth and body size relationships*: *A case study in callitrichid primates*.

**Keegan Selig**.

Assistant Research Professor, Duke University.

*The evolution of mammalian bilophodonty*.

**Amelia Villaseñor**.

Assistant Professor, University of Arkansas.

*Examining the effect of colonially imposed conservation on ecological baselines in biological anthropology*.

Honorable mention:

**Mikel Arlegi**.

Postdoctoral Researcher, Institut Català de Paleoecologia Humana i Evolució Social (IPHES).

*Postural influence on the evolution/co‐evolution of the cranio‐cervical complex in the Neandertal lineage*.

A committee of 27 AABA members, including the Chair, reviewed the pool of eligible proposals. I appreciate the time and effort that each reviewer provided in assessing these applications, and the helpful and constructive feedback given to each applicant. In addition to the Chair, our committee this year included: Benjamin Auerbach, Rebecca Ackermann, Karen Baab, Andrew Barr, Christina Bergey, Aaron Blackwell, Michelle Bezanson, Celeste Gagnon, Christopher Gilbert, Lesley Gregoricka, Mark Hubbe, John Krigbaum, Elizabeth Miller, Connie Mulligan, Charles Musiba, Robin Nelson, Marin Pilloud, EA Quinn, Michelle Rodrigues, Josh Snodgrass, Christopher Schmitt, Nandini Singh, Julie Teichroeb, Erin Vogel, Noreen von Cramon‐Taubadel, and Scott Williams.

### 7.8 Professional Development Panel

The Professional Development Panel for the 2023 AABA annual meeting in Reno will take place on Thursday, April 20, 2023, from 12:15 to 2:15 p.m. The title of the panel is *Let's get Funded*! *Strategies for writing successful grant proposals*. The panel includes panelists from The National Science Foundation, The Leakey Foundation, and the Wenner‐Gren Foundation. This will be a hybrid event, which will be available for viewing through the online AABA meeting platform.

### 7.9 History and Honors Committee

The following report was submitted by Julienne Rutherford.

The primary business of the History and Honors Committee centered on three significant association awards: the Charles R. **Darwin** Lifetime Achievement Award, Gabriel W. Lasker Service Award, and the AABA and Leakey Foundation Communication and **Outreach** Award in Honor of Camilla Smith. Secondary business this year was the implementation of the AABA Oral History Project.

### 7.10 Nominations

Procedures: Using social media, email blasts, the AAPA conference portal, and the AAPA website, we solicited nominations for the Darwin, Lasker, and Outreach Awards. All nominations were due on October 15, 2022. Per the handbook, nominations from the previous year were held over for consideration this year. We required that everyone submitting a nomination also submit a “Nominator's Disclosure Form” asserting that they can attest to the ethical behavior of their nominee.

- Darwin award: 6 nominees for the Darwin award (3 new, 3 rollovers)
- Lasker award: 3 nominees for the Lasker award (2 new, 1 rollover)
- Outreach award: 5 nominees for the Outreach Award: 2 new, 3 rollovers

### 7.11 Voting Process

Nominations were collated for each award and distributed via Google Drive to all voting members of the Executive Committee. This year I implemented rank order voting via Google Form. Voters can rank any number of candidates. For the most part this worked well.

### 7.12 Awardees

- Charles R. **Darwin** Lifetime Achievement Award: **John Fleagle**
- Gabriel W. **Lasker** Service Award: **Andrea Taylor**
- AABA and Leakey Foundation Communication & **Outreach** Award in Honor of Camilla Smith: **Cara Ocobock**

### 7.13 Procedural Issues

For next year, the nomination will be all Google docs/forms: **a one‐page nomination and disclosure form for all signatories. No CVs or additional letters of support**. We received a nomination for a candidate who died prior to voting. We decided as a committee that if an individual who is nominated for an award should pass away before the award voting takes place, they will be removed from consideration for that and subsequent years.

We require the nominators to sign a disclosure form indicating their nominee meets the expectations of the AABA Code of Conduct, the AABA Professional Ethics Policy, and the AABA Statements on Sexual and Other Harassment. As part of our vetting process, which includes the review of nominees by all Executive Committee members, we will ask the H‐CARE and Membership committees to review the nominees against any reports they have received. If the Executive Committee becomes aware of a breach of the expectations outlined in the required disclosure form, even if the nominators were not aware of such a breach, the nominee will be removed from consideration.

#### 7.13.1 Oral History Project

Jon Marks and Karen Rosenberg proposed this prior to the 2022 AABA meeting. After discussion at and after that meeting, I drafted a project and procedure description and disclosure form which were reviewed by Ex Comm and accepted by Jon and Karen. The process includes Jon and Karen proposing a list of potential interviewees to the History and Honors Chair and the Membership Chair, and all interviewers and interviewees completing a disclosure form. The first interview is Friderun Ankel‐Simons, submitted for review by the History and Honors Chair on 4/10/2023 and posting to AABA media platforms ASAP by the Secretary. The plan is to release the week after meetings.

Links to Oral History Project documents/media:

- AABA Oral History Disclosure Form
- AABA Oral History Procedures Document
- Interview with Friderun Ankel‐Simons (running time 1:06:32)

Many thanks to Jon and Karen for their initiative and their patience!

**Other issues/questions** carried over from last year's Ex Comm meeting:

- Where are History and Honors materials archived? Are documents and records just handed over from Chair to Chair?
- Update History of AABA meetings and leadership (circles back to About page)
- Documents at the Smithsonian?
- Proceedings of the meetings (behind paywall)

### 7.14 Student Programs Committee

The following report was submitted by Chelsey Juarez.

The Student Programs Committee primarily implements two student competitions. The first occurs at the annual meeting, when students who are presenting research in either a poster or podium format compete for seven named prizes. The second competition is traditionally for travel funds to attend the annual meeting, for which they write an essay on a topic chosen by the Executive Committee. In this report, I first summarize the 2022 student prizes awarded at the meetings in 2022, and finally, I present the 2023 travel awardees and preliminary information on the 2023 Student Presentation Prize Competition.

#### 7.14.1 2022 Student Presentation Prizes

The deadline for entry to the 2022 AAPA Student Presentation was April 1, 2022. This was the fifth year that we relied on an online submission form. We received significantly fewer student presentation prize applications than in the past (*n* = 21) and 11 judges (Table 9) evaluated these presentations. The 2022 AABA student presentation winners were:

- **Natalie Swinford**. UC Davis. Increased Homozygosity due to endogamy results in fitness consequences in a human population
- **Stephanie Fox**. University of New Mexico. Social relationship quality predicts coalition formation among adult female chimpanzees at Kanyawara, Kibale National Park, Uganda
- **Isis Dwyer**. University of Florida. Limitations of Current Data and Methods for the Forensic Identification of Black Undocumented Immigrants
- **Catherine Kitrinos**. U Mass. Is there a core primate hair microbiome?
- **Mildred Trotter Prize**. Valerie Sgheiza, University of Illinois Champagne Urbana. The effect of demographic variation on correlations between developing teeth in humans
- **Patricia Whitten Prize** Jacob Feder, Stony Brook. Social precursors and fitness outcomes of group fission in geladas
- **The AAA‐AAPA Anatomy in Anthropology podium** Zana Sims and Catherine J Llera Martin, Johns Hopkins University. Using weighted spherical harmonics to detect functional locomotor signals at the distal femoral articulation
- **Journal of Human Evolution Prize on human or primate evolution**. William Callison, Harvard University. Andean populations adapted to high‐altitude hypoxic environments use thoracic ventilation more than lowlanders to breathe while walking and running

**TABLE 9:** History of the number of entries and judges for the student presentation prizes.

Year	Number of entries	Number of judges
2014	39	—
2015	31	—
2016	45	21
2017	69	34
2018	63	39
2019	91	41
2020	53	—
2021	23	16
2022	21	11
2023	30	21

#### 7.14.2 American Association of Biological Anthropologists Honorable Mention for Student Presentations

- *Dori Kenessey*: Identifying candidate SNPs shaping dental morphological trait expression
- *Alexandra Kralick*: Relative leg to arm strength proportions in Bornean and Sumatran orangutans

#### 7.14.3 2023 Pollitzer Travel Awards

The Pollitzer Student Travel Awards are designed to help students defray the costs of attending the AAPA meetings. They are named in honor of William S. Pollitzer, a Human Biologist who taught at the University of North Carolina, Chapel Hill, a Darwin Lifetime Achievement Awardee, and past president of the AAPA. The number of awards is also tied to proceeds from the auction that is held at the annual meeting of the AAPA in the year prior. The award traditionally provides $500 to each recipient to defray travel costs to attend AAPA's annual meeting.

This award is open to all AABA student members (undergraduate and graduate) who are attending the annual meeting. Students do not need to be giving a presentation at the meeting to qualify but they do need to be a member of the AABA at the time of the meeting and should not have been granted their PhD prior to the submission deadline for 2023; the submission deadline was (January 6, 2023).

The essay question changes each year. Awards are made on the basis of an essay of no more than 750 words (excluding references). The specific prompt for 2022 was:

*What does decolonizing biological anthropology mean to you? What steps can be taken to decolonize the research process? The classroom? Field work? What do you think would be the impact of decolonizing these spaces?*

The deadline for entry was January 6^h^, midnight UTC‐11 h. The submission website went live in November 2022 and we received entries starting on the 4th of November through to the last few minutes before the deadline. We received 87 original essays. A committee of 16 judges was convened.

### 7.15 Committee of Judges for the 2023 Pollitzer Travel Award (25 Judges)

Along with the chair, the following members served as judges: Chrisandra Kufeldt, Colleen Cheverko, Evan Garofalo, Krista Fish, Maja Seselj, Mark Hubbe, Scott. Maddux, Addison Kemp, Cris Hughes, Michael Rivera, Melanie Beasley, Jessie Goliath, Nicholas Passalacqua, Teresa V Wilson, Zaneta Marie Thayer, Jada Benn Torres, Eric Bartelink, AnDi Yim, Kelsey Ellis, Melissa Clark, Jonathan Bethard, Heather Edgar, Elizabeth Cho, and Lori Trembly.

Evaluation was done on a 60‐point scale based on a five‐part scale (50 points total for originality and creativity, reasoning based on evolutionary biology and theory, and the current literature of our discipline, including references as needed; and 10 points for grammar and spelling). In an effort to standardize the scoring system, a rubric was utilized.

Each entry was given a number, and essays were identified only by number during the evaluation process. Each essay was scored by three judges. Each judge was asked to score ~8 essays, and one back up judge was used for conflicts of interest. The final score for each essay was determined as the average of the three independent scores. Neither the named essays nor the number/name key was accessible at any time to the judges. No judge evaluated a proposal from a student at their same institution and the chair was not notified of any other conflicts of interest.

In order to implement the policy that priority would be given to novel entrants, students who had received a Pollitzer award previously had 3.5 points deducted from their final score. There were three entries from previous winners. Two of these ranked high enough to win a Pollitzer award again this year even with the penalty.

AAPA had funds to award 30 awards (but two of the individuals attending virtually so we actually had 31 winners!). The winners are listed in Appendix E, and trends in numbers of entries and winners are in Table 10.

**TABLE 10:** History of the number of entries and winners for the pollitzer travel essay competition.

Year	Number of entries	Number of winners
2012	—	43
2013	—	43
2014	—	50
2015	40	24
2016	68	42
2017	118	50
2018	75	57
2019	104	46
2020*	154	50
2021	31	28
2022	40	30
2023	87	30

*Note:* ^*^This meeting was shifted to completely on‐line due to the pandemic.

#### 7.15.1 2023 Student Presentation Prizes

The deadline for entry to the 2023 AAPA Student Presentation was March 24, 2023, and will proceed following the protocol used for the last several years. We received an increase in student presentation prize applications this year compared to last year (*n* = 30) and 21 judges have been organized to evaluate these presentations.

### 7.16 Nominations and Elections Committee

Report submitted by Chair of the Election Committee and President Elect, Leslea J. Hlusko.

This year there was only one elected position to fill. Treasurer Graciela Cabana is moving to be co‐editor of the Yearbook of Biological Anthropology as of the Business Meeting on April 21, 2023. As one person can only hold one position on the Executive Committee, she is resigning her position as Treasurer.

Elections were held to appoint a person to fill the rest of the current Treasurer's term. The elected person will be allowed to run again for a full term as Treasurer, if they want to do so. The election was announced via the AABA Newsletter and by a 19 September 2023 AABA News post on the http://www.bioanth.org website.

Nominations were accepted by email (sent to the Nominations Committee Chair) and through the survey that all meeting attendees are asked to complete at the time of registration and abstract submission. The nomination window closed on October 17, 2022. For all nominations, including self‐ nominations, we required a brief statement explaining how/why the nominee is well‐suited to take on this leadership role. Nominations that did not include this statement were not considered.

Four people were nominated. One declined, another was a student member and therefore ineligible to hold the position as our By‐Laws indicate that only full members hold elected office. The remaining two nominees agreed to stand for election: Jonathan D. Bethard and Michelle Bezanson. They both submitted disclosure forms and candidate bio statements.

Voting opened with the January 2023 Newsletter and closed on February 15, 2023. As specified in the AABA *Constitution and Bylaws*, only Regular members can vote in the election of AABA officers. The link for voting was hosted by Burk Associates. The website authenticated voters as Regular Members, and then permitted submission of votes. Members were required to renew membership in order to vote.

By the time voting closed, a total of 68 Regular Members had voted. Jonathan Bethard received the majority of votes. He was notified by President Steve Leigh on February 17, 2023, and accepted the position.

### 7.17 Committee on Diversity

The following report was submitted by Susan Antón.

Equity, inclusion and community building are truly everyone's work. Although we report here on the formal work of the COD only, we do not mean to diminish the important work being done across the AABA by many constituencies.

The aim of the AABA Committee on Diversity (COD) is to increase diversity, equity, and inclusion within the field of biological anthropology. Established in 2006 and incorporated into the (then) AAPA bylaws as a standing committee in 2011, the COD is a consortium of member‐developed subgroups overseen by an appointed chair and a steering committee. There are now eight (!) formalized interest groups.^4^ We encourage AABA members to join groups that interest them and/or develop missing groups. The overarching COD has a chair appointed by the AABA Executive Committee and a steering committee comprising at least one member from each of the interest groups. The subgroups are organized along standards that are developed independently by each subgroup.

AABA COD is a “bottom‐up” organization and we are excited to recognize a new, and much needed, interest group, COD‐DISABILITY. AABA Early Career Liaison Katie Kinkopf had a vision and has been amazing to work with. See more about Katie's work below.

We are delighted to congratulate this year's AABA Lasker Award winner, Andrea Taylor. Andrea was the Founding Co‐Chair of COD‐WIN. We cannot think of anyone more deserving. Please join the AABA Business Meeting on Friday the 21st to celebrate Andrea!

COD Events at the 2023 AABA Conference.

COD will host a number of activities at the 2023 meeting, both virtual and in‐person. Many are open to all attendees; a few are by invitation. Event details may be found in the subcommittee reports that follow.

*Virtual Events*

- **Tuesday April 4th—**6–8 p.m.—VIRTUAL—AABA Forum and Listening Session on Disability w/COD Disability (open to all)
- **Wednesday April 5th—**noon PST—VIRTUAL—COD URS Undergraduate Research Symposium (open to all)

***In‐Person Events***

- **Wed April 19th—**2:30‐5 p.m. COD WIN Workshop: Experiencing and negotiating the dynamics of power: beginning the conversation (open to all)
- **Wed April 19th—**5‐6 p.m. COD WIN Happy Hour (open to all; cash bar)
- **Wed April 19th—**6‐8 p.m. COD URS Undergraduate Research Symposium (open to all)
- **Thurs April 20th—**8‐9 a.m. COD IDEAS Working Group (Working group members)
- **Thurs April 20th—**10 a.m.−12 p.m. AABA Forum and Listening Session on Disability with COD Disability (open to all)
- **Thurs April 20th—**12‐2 p.m. COD LGBTQQIAA Business Meeting (open to all)
- **Thurs April 20th—**5 p.m. COD IDEAS & COD International Mixer (open to all, cash bar)
- **Fri April 21st—**12‐2 p.m. COD IDEAS Alumni Network Event (open to all)
- **Sat April 22nd**—7‐9 a.m. COD Steering Group—(COD Subcommittee Liaisons)
- **Sat April 22nd**—9‐10 a.m. COD International Committee Brainstorming Meeting (open to all)
- **Sat April 22nd**—10 a.m.–12 p.m. COD‐Disability Workshop: Dialogues and Pathways toward a more accessible Biological Anthropology Open Discussion (open to all)
- **Sat April 22nd**—12:30–2:15 p.m. Committee on Diversity, Committee on Community Partnerships and AABA Presidential Panel: Community Engaged Research (open to all)

### 7.18 COD Works With the AABA Executive Committee and the AABA Early Career Liaison

The COD regularly brings initiatives to the AABA executive committee and likewise serves in an advisory capacity when the President and board seek our input. To this end, the COD Chair attends executive committee board meetings as an ex officio liaison.

In 2022–23 the AABA Early Career Liaison, Katherine Kinkopf, aimed to work with COD to surface issues related to disability and access to the discipline generally and AABA meetings in particular. Over the year she worked on initiatives related to meeting accessibility (you'll have noticed new questions on the registration and abstract forms; that's Katie and AABA Executive Board members) including possibilities for accessing ASL and Simultaneous Translation for some presentations. Katie is a key mover in the Listening Sessions and Workshop on Disability that will be held in Reno. She is the core of the 8th COD interest group, COD‐Disability. Katie Rocks!

The COD Steering Committee (which includes at least one member from each subgroup) proposed several ideas to AABA President Leigh for the Presidential Panel. One of these, *Community Engaged Research*, has been developed as a joint Presidential, COD, and Committee on Community Partnerships Panel. Panelists include COD‐IDEAS members Phoebe Stubblefield, Jada Benn Torres, and Zaneta Thayer as well as others. The panel is Saturday the 22nd at noon.

### 7.19 Highlights of 2022–23 Activities by COD Groups (Mostly in Order of Founding)

*NEW! COD‐DISABILITY* (*est*. *2022; chair Katie Kinkopf*): COD‐Disability's mission is first and foremost to foster belonging and provide a cultural space for disabled biological anthropologists (broadly conceived), to advocate for cultural change in the AABA with regard to disability as an intersectional and politicized identity, as well as to devise long‐term, sustainable, accessibility recommendations to amend the existing AABA poster and presenter guidelines. COD‐Disability is sponsoring two AABA Fora at the 2023 AABA Annual Meetings: a virtual listening session and an in‐person workshop/dialog. (See above).

*COD‐IDEAS* (*est*. *2006; co‐chairs Jada Benn Torres, Agustín Fuentes, Ripan Malhi, and Susan* Antón): COD‐IDEAS held planning and alumni events and a mixer (with COD‐International) at the AABA 2022 Meetings in Denver. Several virtual follow‐up sessions laid the ground work for the submission of a second NSF grant to continue the COD‐IDEAS workshops (previous NSF funding of which has ended). The new NSF submission was made in January (by PIs Graciela Cabana and Zaneta Thayer and Co‐Is Susan Antón and Ripan Malhi). The programming extends and expands the COD‐IDEAS workshop (at the AABA meetings) for developing pathways into biological anthropology, adds regional in‐person COD‐IDEAS workshops in different parts of the country, and adds programming for faculty development through the career lifecycle. Fingers crossed that we will have funding for renewed COD‐IDEAS workshops next year!

COD‐IDEAS continues to sponsor and staff an AABA booth at SACNAS, the STEM Diversity Conference (Sacnas.org). Dartmouth and NYU supported faculty and student participation to staff the booth in 2022. This was the first post‐pandemic, in‐person event featuring the name change, a move that has raised the discipline's legibility to STEM students.

In 2023, IDEAS will reprise the events of AABA 2022 adding a networking challenge to both the Thursday mixer and Friday lunch.

*COD‐URS* (*est*. *2010; co‐chairs Cara Wall‐Scheffler & Marcie Myers*): In 2022, we held our 11th Annual Committee on Diversity Undergraduate Research Symposium (COD URS). We had 32 posters from 26 universities and colleges for the in‐person session. Eleven (34%) of the first‐author presenters are first‐generation college students. Nine of these programs do not have graduate options in anthropology, and three do not offer undergraduate degrees either, so this symposium offered a crucial opportunity for these students to meet and talk with graduate students and potential graduate advisors. We had fantastic representation from across AABA elected officers (all past presidents back to 2012!) and other senior members who came to support the students. We had a further 12 presenters who presented only at the virtual symposium. Seven of the programs represented at the virtual symposium were unable to attend the in‐person session, representing an important option for departments who might not have undergraduate funding to attend conferences. Across all the presentation options, we were able to support a total of 79 undergraduate students in their biological anthropology research.

This year (2023) COD‐URS will again be having both an in‐person and a virtual symposium in order to allow students from a diversity of backgrounds to present their work. The in‐person COD‐URS will be held on Wednesday, April 19 in Reno. We have accepted 49 posters representing 34 labs for the in‐person session. We have four presenters who will be presenting only at the virtual symposium (noon PST on Wednesday, April 4), although we expect many of the other presenters to attend as well. Eleven of the fifty‐three first authors (21%) are first‐generation college students, and at least ten of the 34 labs do not offer advanced degrees in anthropology. Across all the presentation options, we are able to support a total of 81 undergraduate students in their biological anthropology research.

*COD‐WIN* (*est*. *2013; co‐chairs Michelle Bezanson and Anne Stone*): The subcommittee held virtual committee planning meetings and hosted an interactive panel discussion at the 2021 AABA meetings. The panel was titled: “Mentoring for Mental Health”. Mental health expert Nicole Banks and Michelle Bezanson were the main speakers and 21 AABA members at all career stages attended the workshop. It was a very successful event with most participants wanting more. Sadly, we only received six responses to our post‐workshop survey, but these were positive and informative. We have had several virtual meetings to discuss the 2023 COD‐WIN event: *Experiencing and negotiating the dynamics of power*: *Beginning the conversation*. The event will be held Wednesday April 19th 2023, in person.

In 2022–23, the following new committee members have been invited and will join COD‐WIN: Meghan Holms, Stephanie Poindexter, and Rebecca Rogers‐Ackermann.

*COD‐LGBTQQIAA* (*est*. *2013; steering committee liaison Stephanie Meredith*): COD‐LGBTQQIAA held their business meeting at the 2022 AABA meetings, during which time the group resolved to explicitly make connections with some of the other identity‐based minority COD‐related groups and several small groups of people connected to collaborate on bringing forth topical invited sessions and workshops at future meetings. To that end, Austin Lawrence and Chris Stantis hosted a January “*Bite‐sized Bioanth*” Zoom meeting in which Isis Dwyer from the IDEAS scholar program presented her research, and Samantha Archer and Alexandra Kralick put together an excellent invited symposium on “*Queering research*: *uses of LGBTQIA+ perspectives and queer theory in biological anthropology*.”

*COD‐AACT* (*est*. *2013; chair Jessica Westin*): COD‐AACT is regrouping. The subcommittee has an open discussion meeting at the AABA (see above). If you identify with or are interested in any part of our acronym (non‐anthro department or teaching‐heavy positions, contingent or contract faculty assignments), please attend our meeting to discuss ideas for workshops, panels, sessions or anything else for future meetings and the AABA in general. Ideas may also be sent to current chair Jessica Westin.

*COD‐International* (*est*. *2014; chair Rebecca Ackermann*): The International Scholars group is co‐hosting a mixer with COD‐IDEAS on Thursday night, and will hold a brainstorming meeting on Saturday morning. Please join us!

Interested in starting another COD subgroup? Please contact Susan Antón the COD Chair.

Interested in joining a subgroup? Please contact the subcommittee Chair or attend their events.

### 7.20 Education Committee

Submitted by Drs. Kate McGrath and Rob O'Malley, co‐chairs.

#### 7.20.1 2022 Conference‐Related Activities

As part of the AABA 2022 Annual Meeting the committee hosted a teacher workshop at the Denver Museum of Nature and Science (DMNS) on Saturday March 26 2022. The half‐day workshop on “Teaching human and primate evolution in middle and high school classrooms” was attended by 16 educators and included brief presentations and conversations by Dr. Briana Pobiner, Dr. Rob O'Malley, Dr. Kate McGrath, Dr. Kerryn Warren, and Rose Leach Basom, with a focus on classroom‐ready resources, activities, and approaches. We received very positive feedback from attendees and our DMNS hosts, who shared afterwards that the workshop provided “the perfect blend of theoretical framework and practical tools for the educators.”

Leading up to the 2022 meeting, the committee greatly expanded the website content for our section of the AABA website (https://bioanth.org/about/committees/education‐committee‐page/) to include definitions and frameworks for public engagement with science, a broad array of resources (including funding opportunities and training materials), lesson plans for biological anthropology content aligned with NGSS, and examples of public science communication and engagement by AABA members. Dr. Katrina Yezzi‐Woodley presented a poster on the Education Committee's history, ongoing activities and resources, and future plans. Finally, Drs. Kate McGrath and Rob O'Malley were named new co‐chairs of the committee, succeeding Dr. Briana Pobiner who chaired the committee since 2016. The entire committee is grateful for Dr. Pobiner's outstanding leadership for so many years!

#### 7.20.2 2023 Conference‐Related Activities (Planned or Underway)

*Internal and external funding support*: In response to AABA leadership's call for funding requests at the 2022 AABA business meeting, we made a formal request for 3 k to support the committee's engagement activities. We have used or will use this support for covering printing costs associated with engagement work, expanding our cast collection, covering AABA meeting catering, and travel related to local outreach activities. We are very grateful for AABA's support! The committee also wishes to gratefully acknowledge the Leakey Foundation for providing small honoraria for AABA members who are taking time out of the meeting to participate in outreach activities.

*Public engagement training*: Dr. Briana Pobiner will provide a virtual training session on science engagement fundamentals for committee members on April 4th, sponsored by the Leakey Foundation. **Workshop support**. We are using AABA funding support to provide light catering for two workshops proposed or co‐proposed by committee members and aligned with the education committee's mandate—Up Goer Five: Using Simple Language to Communicate Your Research to the Public (Wednesday April 19th, 1–4 p.m.) and Improv for Anthropologists (Thursday April 20th, 6–8 p.m.). Committee members are also individually boosting the workshops within their networks and on social media.

*Outreach*: The Education Committee will host an engagement table at The Discovery (Reno's local science center) on the afternoon and evening of Wednesday April 19th. Participants will make use of the cast collection, video clips, and print resources in short activities to share with and learn from visitorsabout biological anthropology. After the AABA meeting, the committee's (newly expanded) cast collection will be left with the AABA local arrangements committee for use by local educators who may wish to use it in classrooms or other science learning activities.

*On‐site engagement*: We will pilot a small outreach effort at the Peppermill Resort itself, exploring whether venue guests and staff might be an audience for public engagement at this and future AABA meetings. We will have a small table, posters, casts, and AABA education committee members set up near the registration desk on Thursday April 20th from 12 to 1:30 p.m.

*Committee meeting*: An in‐person/virtual committee meeting will be held on Saturday April 22nd from 8 to 9:30 a.m. We hope to plot out some specific, actionable goals for the coming year and recruit new members from AABA into the committee.

#### 7.20.3 Other Committee Efforts

The committee has hosted four virtual meetings since the 2022 AABA meeting. Committee efforts underway or planned for the next year include:

*Formalizing committee roles*: To recognize and incentivize work on behalf of AABA, we are formalizing committee roles beyond the committee co‐chair roles (e.g., “website coordinator”, “resource developer”). This will provide committee members a more specific title they can list on their CVs, tenure documents, grant proposals, etc. beyond “committee member.”

*Refinement of website content and lesson plans*: The committee is working to streamline the process for updating/revising workshop content. At the AABA meeting we plan to advocate for a more prominent link from the AABA landing page to highlight committee content. We are updating some lesson plans that are already online to align with changing vocabularies and frameworks.

*Survey of AABA membership*: The committee has requested permission from AABA leadership to release a survey to AABA members during the 2023 meeting, to gauge current awareness of committee activities and materials, and to hear from AABA members about what they would like to see from the committee over the next few years.

*Explore the feasibility and structure of a public engagement/education awards or small grant program* to recognize, incentivize and support such work by AABA members (and particularly early career members). This is envisioned as a complement to the existing AABA and Leakey Foundation Communication and Outreach Award in Honor of Camilla Smith.

*Expand public outreach efforts in collaboration with the Leakey Foundation*: We will continue to organize and conduct outreach in K‐12 school or museum settings at future meetings. We will also explore how we (or AABA members at large) can participate in or organize virtual engagement opportunities in collaboration with the Leakey Foundation.

#### 7.20.4 Active Committee Members as of March 2023

Active committee members have attended at least one meeting or activity of the AABA Education Committee since April 2022. They include Kate McGrath, Rob O'Malley, Karol Chandler Ezell, Briana Pobiner, Samantha Porter, Kinley Russell, Caitlin Schrein, Katrina Yezzi‐Woodley, Malorie Albee, Amanda Owings, Marin Pilloud, Jacqueline Garnett, Kristin Crouse, Kaitlin East, Arielle Johnson, Seth Chagi, Amanda Ellwanger.

### 7.21 Ad Hoc Committee for Community Partnership

Submitted by Benjamin Auerbach.

The Committee for Community Partnership (CCP) for the AABA has a current membership of four individuals: Drs. Benjamin Auerbach (chair), Shamsi Berry, Ellen Lofaro, Ripan Malhi. Dr. Charlotte Roberts resigned from the Task Force and the CCP in May of 2022. Herein we report our activities over the last year and plans for the upcoming year. In brief, we have been active as part of the Task Force for the Ethical Study of Human Remains, while also serving in our primary role to give advice when requested by the Executive Committee concerning community partnership in research. We ask the EC to consider approaches we outline below about the use of human remains in teaching. In addition, we also have requests concerning the membership and future of this important committee within the AABA.

#### 7.21.1 General Activities in 2022–2023

As noted in the 2022 committee report, after the formation of the Task Force leadership at the request of President Steve Leigh and the Executive Committee, members of the CCP were asked to participate in the Task Force given their broad experience and knowledge as members of the committee. All agreed to serve as members at the time, and all but Charlotte Roberts continue to serve on the Task Force. The Task Force reported its preliminary report in November 2022, and is currently working toward finishing its main goals by the end of 2023.

The CCP was asked in early 2023 to co‐sponsor the Presidential Plenary Panel at the Annual Meeting, which the committee members quickly agreed would be a good idea. With Susan Anton, chair of the Committee on Diversity, Ben Auerbach worked to identify individuals to serve on the plenary panel, which is focused on community partnered and engaged research.

#### 7.21.2 Approaches to Advising the AABA Community About Human Remains in Instruction

Concerns about the use of Native American human remains in instruction, as well as continued questions about both research and instruction using remains without consent by donors or communities, have been brought to the attention of members of the committee with regularity. In light of this, members of the committee ask the Executive Committee to be proactive within the AABA community in reminding individuals and institutions about the proper procedures for working with human remains under NAGPRA. We also encourage the Executive Committee to update the public‐ and member‐facing advice on NAGPRA. Until the work of the AABA Task Force and sibling task forces and commissions at other organizations is complete, we also askthat the AABA highlight examples of research that demonstrate good community partnership. (Such examples would not necessarily endorse this research, and the committee does not want individuals to look at such studies as a series of boxes to check. Rather, these will help researchers see examples of well‐considered and engaged approaches to working with communities.) The committee is available to help in all these endeavors and hopes we can address them in the near term.

We especially note concerns raised about the use of human remains of unknown provenance in teaching, as well as a desire to have clear consent for the use of human remains by at least some individuals in the AABA professional community. We ask the Executive Committee to consider ways to advise the community about how best to address these concerns when they fall outside of existing repatriation law. One suggestion from the committee is that the AABA consider ways to leverage existing donated collections to become sources of human remains for teaching osteology.

#### 7.21.3 Requests About the Future of the Committee Membership and Status

Given the work on the Task Force and delays brought by the pandemic, Ben Auerbach will stay on, with the Executive Committee's approval, as chair until the annual meeting of the association in 2024. We ask that one of the existing members of the committee be allowed to replace him as the committee chair, with a term to be decided between that individual and the EC, unless the committee is transformed into a standing committee (see below).

With Executive Committee approval, the committee is looking toward finding two new full members to rotate onto the committee as soon as possible this year. We would like to recruit two junior scholars with experience in community partnered/engaged research to serve on the committee, potentially asking either individuals who are serving on the Task Force or those who are participating in the 2023 Presidential Plenary Panel in April. The committee would also like to recruit a student member.

Finally, given the growing importance of community partnered research going forward in all work among biological anthropologists, the committee formally requests that the Executive Committee seek an amendment to the AABA Bylaws to change the Committee for Community Partnership into a standing committee.

Thank you all, again, for your continued support of the efforts of our committee. We greatly appreciate the hard work of the members of the Executive Committee.

## 8 AABA Ad Hoc Harassment Committee for Awareness, Response and Equity

The following report was submitted by Andrea B. Taylor.

The HCARE was established by the AABA Executive Committee leadership in February of 2020 as an ad hoc committee. The committee members serve by appointment of the AABA President. The committee is overseen by a chair, who is appointed for a four‐year term by the AABA President. Committee members include Jessica Brinkworth, Agustín Fuentes, Stephanie Meredith, and Robin Nelson.

The HCARE was charged with the following responsibilities:

1 Assisting the Executive Committee officers in creating, implementing, and revising harassment, bullying, intimidation, retaliation, and/or assault reporting policies and procedures.
2 Receiving and responding to incoming reports of harassment, bullying, intimidation, retaliation, and/or assault.
3 Deliberating and determining appropriate responses.
1 Making recommendations to the AABA President.

The HCARE met seven times between March 31st, 2022 and March 31st 2023.

### 8.1 Complaints

The HCARE received and assessed two written complaints regarding concerning behaviors. Both complaints were communicated to HCARE via aapahcare@gmail.com. One of the two complaints involved concerning behaviors that occurred at an AABA annual meeting.

I extend my sincere gratitude on behalf of the HCARE to the AABA Executive Committee for their generosity of time and spirit and their unwavering support of the work of this Committee.

## 9 Reports by Affiliated Organizations

### 9.1 American Association for the Advancement of Science (AAAS) Affiliates and Section H‐Anthropology Updates

The following informal report was submitted by George R. Milner, Section H Secretary.

I should start by thanking Steve Leigh for inviting me to report on what is happening in Section H, Anthropology, of the American Association for the Advancement of Science (AAAS). Unfortunately, I could not attend the AABA meeting this year, so Steve is also presenting this report.

The current year—2023—is a period of major transition for the AAAS, and therefore Section H. This is the first year of the organization's new governance structure. It has been several years in the making, and one of our AABA members, Karen Strier, was heavily involved in that process.

The new AAAS structure is designed to increase governance transparency, to ensure the organization becomes more inclusive, and to recognize and represent today's diverse scientific workforce. It is the second major governance change since the founding of the AAAS in the 19th century. The previous change took place about 70 years ago.

Needless to say, the AAAS from top to bottom is currently feeling its way through this process. For the sections, the immediate concern is to put into practice the intended roles of an expanded group of section officers who started their terms on January 1, 2023. Over the next couple of years, elections will be held for each of the positions, shingling in people to maintain continuity.

The AABA is well represented in the current AAAS officers. In fact, the majority are AABA members. For example, Anne Stone is Chair and Susan Antón is Council Delegate. If anyone in today's audience is also an AAAS member and is interested in running for office, I encourage you to contact either Anne or me.

In 2022, I am pleased to say that 11 new Section H Fellows were named, with AABA members being well represented among them. Our 2023 Robert W. Sussman Award went to Sheela Athreya. To quote from the announcement, she “exemplifies the spirit of this award with her outstanding contributions to understanding the evolution of the genus *Homo* in the middle and late Pleistocene, especially with in Southern and Eastern Asia, and with her holistic approach to investigating variation within and across populations.” The section is pleased that the outstanding achievements of so many of our colleagues have been recognized over the past year.

I'll close by saying that next year's AAAS meeting will be in Denver. I look forward to seeing many of you there.

### 9.2 National Science Foundation (NSF) Program in Biological Anthropology, Division of Behavioral and Cognitive Sciences, Directorate of Social, Behavioral, and Economic Sciences (SBE)

The following report was submitted by Dr. Rebecca Ferrell, Program Director, Biological Anthropology.

The Biological Anthropology program at the National Science Foundation (NSF) continues to support doctoral (DDRIG) and faculty (senior) research on topics spanning our discipline. In 2022, the program published a revised DDRIG solicitation as well as a new senior solicitation that includes new funding opportunities (see below) and requirements.

The program's budget has remained relatively flat in recent years. DDRIGs have remained steady at around 30–35 awards and $800 K per year. The annual number of senior awards has decreased slightly as the cost of research increases. Details of active awards funded or co‐funded by Biological Anthropology can be found here: Bio Anthro Active Awards as of 4/14/23.

As reported last year, the program recently joined a cross‐directorate funding opportunity, Mid‐Career Advancement (MCA), and has made two MCA awards to date. Researchers from our community also have been successful in obtaining support from other NSF programs, including the Build and Broaden, SBE Postdoctoral Research Fellowship, Ethical and Responsible Research, and Graduate Research Fellowship programs and core programs in other research directorates.

The program thanks the biological anthropology research community for their support of the peer review process through service as panelists and ad hoc reviewers. If you are interested in serving in this capacity and are post‐PhD, please send an email with CV or expertise keywords to: anthroreviewerinterest@nsf.gov and/or contact the Graduate Research Fellowship Program (htps://nsfgrfpreviewers.org).

The program has benefited immensely from the addition of a rotator program director position. The search for the next rotator is underway as the current rotator, Dr. Robin Bernstein, will be returning to her home institution later this year. I want to thank Robin for her collegiality, scientific insights, and dedication to the program and to broadening participation in our field. She has been particularly instrumental in expanding the reviewer pool and helping to craft the new and revised program solicitations (see details in Appendix F).

### 9.3 2023 Student Liaison Report

The following report was submitted by Dori Kenessey.

#### 9.3.1 Committee Structure

As per the structure created by the Executive Committee of the American Association of Biological Anthropologists (AABA), the ad hoc Student Committee co‐chairs are the current and incoming Student Liaisons for the AABA Executive Committee. The current (and outgoing) liaison is Dori Kenessey. The incoming liaison is Elise Adams.

#### 9.3.2 Student Liaison Activities

- The eighth annual AABA Student Mixer is scheduled for Wednesday, April 19, 2023. It will be held in Naples 3 of the Peppermill Resort Spa Casino. Considering the ease in COVID restrictions, most student members are expected to attend the 2023 conference in person. Therefore, an additional mixer is not currently scheduled for the virtual setting of the conference (the 2023 conference is held both in person and virtually). The mixer will involve a networking activity to encourage student discussion. Completion of the activity will come with a small reward.
- The AABA Student Members Facebook group has been suspended. This was a previous forum for students to interact, ask questions, receive notification of field school and workshop opportunities, and share current research in the field. The deactivation of this group was caused by COVID and the misuse of the group by members. There has been additional discussion about the suitability of Facebook as a preferred social media platform by the new generation of students. Currently, there is no forum to facilitate student member discussion and the liaison has no effective way to communicate with student membership. Establishing a new method of student communication will be raised by the Student Liaison at the 2023 AABA Executive Meeting.
- Several student programs and efforts established pre‐COVID have fallen through the cracks due to quarantine. With in‐person meetings reinstated, whether to bring these programs back needs to be evaluated. These programs include the “AAPA Room Sharing” group, the Buddy Program, the Student Newsletters, the ad hoc Student Committee meeting, the AABA Student Mixer and discussion panels.
- During and directly after COVID, the virtual and hybrid nature of the meetings has caused difficulties in the liaison transition process. Once the student programs to continue post‐COVID are agreed upon, a formal, itemized document (e.g., handbook) listing all responsibilities that come with the role of AABA Student Liaison should be created to facilitate the transition process. Such a document will provide clear guidance on Student Liaison roles and responsibilities. Should these roles change, the document can be revised with approval by the Executive Committee.

#### 9.3.3 Planned 2023 AABA Meeting Student Committee Events

- AABA Student Mixer: Wednesday 5–6 p.m., April 19, 2023

## 10 Remembering Members and Friends of the AABA

The *in memoriam* report was prepared by President‐Elect Leslea Hlusko. Colleagues recognized included Yves Coppens, Wendy Dirks, Fabien Génin, Bill Jungers, Bill Kimbel, Kamoya Kimeu, Judith Masters, Paul Mellars, Stephen Ousley, M. Kathleen Piterri, Sebastian Ramirez Amaya, Steve Ross, Chuck Snowden, Elanor Sterling, and Stuart McKee Struever.

## 11 Old Business

Remaining Kansas State documents revised to complete the name change to the American Association of Biological Anthropologists.

## 12 New Business

The AABA will draft a statement in support of gender and sexual minorities, and will schedule related workshops and listening sessions in the coming months.

The Local Arrangements Committee for Los Angeles, California, 2024 shared information on the venue and site (Kristi Lewton and Stephanie Meredith).

The meeting was adjourned at 8:45 p.m. Pacific Daylight Time by Steve Leigh.

## Author Contributions

**Amy L. Rector:** writing – original draft (equal), writing – review and editing (equal).

## Data Availability

All data and reports are also available on the AABA website, bioanth.org.

## Endnotes

^1^
Since 2017, AABA finances have been tracked using an accrual accounting system. Unlike a cash‐based system, income and expenditures are recorded when incurred, meaning that transactions are recorded when they are made, regardless of when they are paid. For example, income generated in the Fall of 2020 for 2021 membership would not be considered 2020 income; the recording of this income is deferred until 2021.

^2^
Starting in 2020, the AABA began transferring 3.5% of its investment income into its operating account on an annual basis.

^3^
The regular membership voted to increase regular membership dues by 30% at the 2019 Business Meeting; this went into effect in 2020.

^4^
Committee on Diversity Increasing Diversity in Evolutionary Anthropological Sciences (COD‐IDEAS); COD Women's Initiative (COD‐WIN); COD Undergraduate Research Symposium (COD‐UGRS); COD LGBTQQIAA interest group; COD Anthropologists Outside of Anthropology Departments, Contingent, and Teaching Focused Faculty (COD‐AACT) Task Force; COD International (COD‐I); COD Transforming Anthropological and Scientific Knowledge (COD‐TASK); COD Disability.

## Appendix A 2022–2023 Timelines

**Table:**

Event/process	Open date	Close date	Reviews complete	Notification date
Program committee formation	5/23/2022	7/8/2022	NA	7/17/2022
Symposium proposals	7/15/2022	8/15/2022	8/31/2022	9/9/2022
Abstract submission	9/15/2022	10/17/2022 deadline, 10/26/2022 assigned to reviewers	11/23/2022	12/1/2022 (reject), 12/5/2022 (accept)
Workshop proposals	9/30/2022	11/15/2022	12/17/2022	12/17/2022
Presentation schedule				2/10/2023

## Appendix B 2022–2023 and Historical Meetings Metrics

**Table:**

	2018	2019	2020	2021	2022	2023
	Austin	Cleveland	Los angeles (canceled)	Baltimore	Denver	Reno
Registrations	1901	1802	NA	1079	1281	1250 (as of 4‐3‐2023)
Abstracts submitted	1229	1121	1247	458	785	775
Abstracts accepted	1214	1090	1226	457	776	772
Symposia (submitted/accepted/presented)	26/26/24	23/22/21	26/25/24	8/8/8?	13/13/13	13/13/13
Podium symposium sessions	8	7	6	NA	6	5
Poster symposium sessions	16	14	18	NA	5	6
Online symposium sessions	NA	NA	NA	8	2	2
Contributed sessions	51	41	46	70	85	58
Podium sessions	22	18	23	NA	18	21
Poster sessions	29	23	23	NA	18	20
Evening poster sessions	2	1	0	NA	0	0
Online sessions	NA	NA	NA	70	49	16
Withdrawn submissions (as of 4‐16‐2023)	30	16	11	5	17	23

## Appendix C International AABA Registrants by Country, 2019–2023

**Table:**

Country	2019	2021	2022	2023
Argentina	1			
Argentina	1	5	4	3
Australia	6	7	7	7
Austria	2	4	5	4
Belgium	4	2		3
Brazil	16	6	4	3
Canada	74	51	34	46
Chile	l	1		2
China	23	2	1	3
Colombia	2	l		4
Croatia	3	l		3
Cyprus	l			
Czech Republic	5	1	2	5
Denmark	5	2		2
Egypt, Arab Rep.	1			
Estonia	1			
Ethiopia	2			
Finland	4			
France	17	4	l	2
Germany	21	14	14	14
Greece	l			5
Hong Kong SAR, China	l			1
Hungary				1
Iceland	1			
India	2	3	2	2
Iran., Islamic Rep.	1			
Israel	2	3		5
Italy	2	2	2	6
Japan	4	4	2	3
Kenya	1			
Korean Rep.	1	2		
Lebanon	1			2
Malaysia				1
Mexico	4	12	4	3
Netherlands	3	1	4	
New Zealand	7	5	1	1
Nigeria	2	2	1	
Pakistan	1			
Panama	1	1		1
Peru	2			
Philippines				1
Poland	3	3	4	2
Portugal	1			1
Puerto Rico				1
Russian Federation	2	3		
Singapore	1			1
South Africa	4	18	12	11
Spain	11	5	8	11
Sweden	1	1	1	
Switzerland	4	8	6	7
Tanzania	1			
Thailand				1
Uganda	2			
United Kingdom	60	27	35	26
Total count	301	201	170	195
% of registrants	17%	19%	15%	18%

## Appendix D Member Applications

**Table:**

*New special members* (16)	
Maya Alibrio	Diane Gelvin
Eric Anderson	Elizabeth Hannigan
Kayla Bellipanni	Leigh Hayes
Seth Chagi	Elena Krane
Nathan Cooley	Meisshialette Ortiz
Hugh Dyar	Thomas Rohrer
Taylor Fisher	Catherine Alice Ramos
*New Regular postdoc/contingent members* (33)	
Francisca Alves‐Cardoso	Sarah Reedy
Kristine Beaty	Giulia Riccomi
Laura Bolt	Carolin Röding
Joe Califf	Jessica Ryan‐Despraz
Meadow Campbell	David Schruth
Eve Cooper	Alexandra Schuh
Flavio De Angelis	Laurits Skov
Constanza de la Fuente	Kaitlyn Stiles
Jenna Dittmar	Teresa Stoker
Christianne Fernée	Jessica Stone
Janeal Godfrey	Ian Towle
Claire Hodson	Tauras Vilgalys
Angeline Leece	Xueye Wang
Marina Martínez de Pinillos	Ashleigh Wiseman
Mario Modesto Mata	Arslan Zaidi
Sarah Phillips	Elena Zavala
Amber Plemons	
*New regular members* (45)	
Christopher Aris	Tamás Hajdu
Ron Barrett	Casey Holliday
Carol Berman	Michelle Hughes Markovics
Melinda Carter	Ammie Kalan
Celise Chilcote‐Fricker	Harvinder Kaur
Jennifer Clark	Terry Kensler
Loren Davis	Katharine Kolpan
Florent Detroit	Marta Krenz‐Niedbala
Christine France	Natalie Langley
Macarena Fuentes‐Guajardo	Jorge Lazareff
Alexandra Greenwald	Xiaoming Liu
Aryeh Grossman	Sylwia Lukasik
Nasser Malit	Myron Shekelle
Lauren Minford	Sarah Stark
Tesla Monson	Kohei Tamura
Gillian Moritz	Nicole Thompson Gonzalez
Liv Nilsson Stutz	Sylvere Valentin
Christina Papageorgopoulou	Vivek Venkataraman
Gabriel Popescu	Linda Vigilant
Ben Potter	Melissa Wilson
Maanasa Raghavan	Aram Yardumian
Elizabeth Sawchuk	Rene Zielinski
India Schneider‐Crease	
*New student members* (264)	
Brianna Abba	Devyn Caldwell
Amaya Abeyta‐Brown	Sarah Caminito
Belkis Abufaur	Hannah Carson
Kara Adams	Julia Casorso
Devin Adcox	Mortimer Cavanah
Brent Adrian	Bridget Chak
Asterios Aidonis	Clara Chamorro
Laura Allen	Elisabeth Chaumont‐Sturtevant
Meredith Amato	Coral Chell
Nayantara Arora	Tara Choat
Emma Askren	Kristine Joy Chua
Daniel Aslan	Jillian Clarke
Bruno Athie Teruel	Celia Cleary
Denise Axume	Savannah Cobb
Juliet Aze	Blair Coe Schweiger
Carolina Baez	Cheyenne Collins
Lydia Bailey	Miguel Contreras Sieck
Madeline Ballinger	Mycahll Cook
Esha Bandyopadhyay	Mitch Cook‐Graver
Ariel Barrera	Cesar Cornejo Ochoa
Tyler Barrett	Sara Cotton
Ian Barry	Ariel Creal
Elizabeth Beachem	Diana Cruz Dávalos
Juliette Bedard	Jessica Czapla
Bobbie Benavidez	Ailis Daly
Sarah Borgel	Cassandra Davis
Robert Boyce	Rosseirys De La Rosa
Rebecca Brittain	Luca Del Giacco
Kristen Browne	Thomas Delgado
Janeth Cabanas	Riley Derby
Cai Caccavari	Anelis Diaz
Larra Diboyan	Jordan Hoffman
Daniel Dillon	Te Wai Pounamu Hona
Juliana D'Orazio	Veronica Hong
Katherine Dunning	SaMoura Horsley
Sandy Durden	Lily Hou
Claire Eichen	Kaitlin Hsu
Marcy Ekanayake‐Weber	Jinguo Huang
Melinda Elser	Madison Hubbart
Angelina Emmons	Veronica Hughes
Sedona Epstein	Madison Hull
Meg‐Kyla Erasmus	Tabasom Ilkhan
Marisol Espitia	Spencer Irvine
Stephanie Fannin	Kendra Isable
Casey Farmer	Shelby Jarrell
Michelle Fleishman	Christopher Johnson
Samantha Foppe	Julia Johnston
Emilia Franklin	Brooke Jones
Lucia Freedberg	Jasmine Juette
Kiara Frey	Kamryn Keys
Sarah Friesen	Chloe Knitt
Stephanie Fuehr	Matthew Koelbel
Elissavet Ganiatsou	Lauren Koerner
Guoshuai Gao	Amelia Konda
Elijah Gardner	Krystal Koski
Kalila Garrison	Daniël Kotze
Angeliki Georgiadou	Rylee LaLonde
Will Gerardo	Chloé Lauer
Madeleine Getz	Jennifer Laughton
Delaney Glass	Adam Law
Aliya Godoy	Zaira Leal
Louis Gorgone	Alice Lee
Emmaleigh Grady	Sol Lee
Carly Green	Mary Lerch
Ethan Gulledge	Jenna Levine
Logan Guthrie	Hannah Liedl
Meghan Hanson	Sheily Lim
Taylor Harman	Amanda Lima
Jeffrey Harris	Angelique Lindberg
Lauren Hayden	Jenny Lu
Laura Haynes	Sabrina Maccaron
Kristy Henson	Maïa Magalhaes‐Filion
Madelyn Hertz	Megan Malherbe
Milan Hilde‐Jones	Clara Mariencheck
Elena Hinz	Benjamin Martin
Erika Matthews	Weldeyared Reda
Winfred Mbogo	Arielle Reich
Jenneca McCarter	Aleisha Reynolds
Phillip McCheyne	Miles Rhoads
Steph McClennon	Alicia Rice
Katie McIlhennymiller	Smarika Rijal
Kayley McPhail	Milena Romano
Alanna Melchor	Marco Romboni
Tempest Mellendorf	Chelsea Rose
Ashley Mensing	Irmine Roshem
Arianna Method	Zachary Rude
Trace Miles	Daniel Runty
Cambria Miller	Alexandria Sambrano
Daniela Mirabal Torres	Livia Santos
Maria Fernanda Miron Toruno	Abby Sargent
Miriam Miyagi	Mia Sarkisian
Maren Moffatt	Paige Schmitt
Anuska Mohapatra	Rena Schwartz
Calvin Mole	Noah Scruggs
Christina Moore	Sarah Seeley
Patricia Morales Lorenzo	Yael Shekel
Brooke Morris	Mark Simon
Daria Moser	Robin Singleton
Miranda Mueller	Anastasia Slack
Shubhra Murarka	Kyle Smith
Hannah Nairn	Sarajane Smith
Robyn Nakano	Harshal Sonekar
Sharifah Namaganda	Abigail Sorenson
Nathan Nguyen	Angelos Souleles
Lauren Nichols	Cristina Stan
Ceara Nicholson	Kathleen Stansbury
Judyta Olszewski	Nadine Steer
Maire O'Malley	Diego Stokes‐Malave
Everlyne Omondi	Kristen Stout
Christianne Ormsby	Samar Syeda
Leslie Paige	Christine Ta
Magdalena Palisson	Rosa Taylor
Sarah Patterson	Indya Thompson
Laura Perez	Soňa Tökölyová
Jeannine Pili	Ursulina Tomczak
Paige Plattner	Abbigail Tondryk
Brooke Priest	Malachi Tripaldi
Putu Pujiantari	Kristen Tuosto
Sheana Ramcharan	Kirsten Twork
Michelle Txarola	Megan Walsh
Jose Ubeda	Mykala Ward
Oriasotie Ujaddughe	Lindsey Warshawski
Emma Ulrich	Xuan Wei
Muneebah Umar	Cole White
Massiel Valcarcel	Kerri Widrick
Lauren Vamos	Sara Williams
Alexander Vasquez	Sterling Wright
IJk van Hattum	Samantha Yawitz
Katherine Velasquez	Carlotta Zeppilli
Edgar Villeda	Stefania Zingale
Chelsey Voegt	Ruiqi Zou
Madelyn Wachnuik	Veronica Zubritzky
*Student member to regular post‐doc/contingent member* (23)	
Malorie Albee	Paige Madison
Kevin Bird	Lauren Meckel
Elizabeth Cho	Selim Natahi
Carter Clinton	Enquye Negash
Cecilia Collins	Deborah Neidich
Kristin Crouse	Robert Patalano
Rong Fan	Rachel Petersen
Frederick Foster	Taylor Polvadore
Deanna Goldstein	Leslie Quade
Alexana Hickmott	Kathleen Reinhardt
Kelly Kamnikar	Florian Trebouet
Justin Lund	
*Student member to regular member* (20)	
Olumayowa Adegboyega	Catherine Llera
Armando Anzellini	Courtney McConnan Borstad
Derek Boyd	Alyssa Newcomb
Elizabeth Clausing	Kaela Parker
Elizabeth Dinkele	Christopher Rainwater
Felix Engel	Sarah Rosen
Lori Fields	Caroline Rowe
Emma Finestone	Heather Shattuck‐Heidorn
Haley Horbaly	Travis Steffens
Stephanie Levy	Victoria Swenson
*Student member to special member* (5)	
Danielle Carmody	Matthew Rolland
Esther Choo	Ayesha Tarek
Sophia Reck	
*Special member to student member* (4)	
Julia Stone	Paula Monguí
Kayla Genord	Kenneth Tremblay
*Regular post‐doc/contingent member to regular member* (2)	
Julie Fleischman	Shelby Putt
*Regular member to retired member* (2)	
Julia Beaumont	Bruce Gelvin
*Special member to regular member* (2)	
Carrisa Pritchard	Michelle Tabencki
*Special member to regular post‐doc/contingent member* (1)	
Xenia Kyriakou	

## Appendix E 2023 Pollitzer Student Travel Award Winners

*2023 Pollitzer Travel Award Recipients*:

Jordie Hoffman, University of Calgary.

Laura Hunter, University of Chicago.

Daniela Orozco, The National Laboratory of Genomics for Biodiversity.

Magdalena Palisson, Pontificia Universidad Católica de Chile.

Dayanira Lopez, University of Nevada Las Vegas.

Kyle Smith, The Pennsylvania State University.

Lydia Bailey, University of Pittsburg.

Staphanie Fannin, The Ohio State University.

Sharmi Sen, University of Michigan.

Te Wai Pounamu, University of Queensland, Brisbane, Australia.

Carlotta Zeppilli, Sapienza University of Rome.

Krista Bennett, Louisiana State University.

Robin Singleton, University of Oregon.

Celia Cleary, George Mason University.

Siobhan Summers, University of Florida.

Madeleine Kelly, University of Chicago.

Maria Sellers, George Mason University.

Laura Pott, University of New Mexico.

Isis Dwyer, University of Florida.

Lindsey Warshawski, University of Victoria.

Rosa Taylor, University of Oregon.

Megan Malherbe, University of Zurich.

Jana Valesca Meyer, University of New Mexico.

Lucia Freedberg, UC Santa Barbara.

Andrea Zurek‐Ost, University of North Carolina.

Liam Johnson, University of Nevada Las Vegas.

Jaime Zolik, University of New Mexico.

Kaita Gurian, The Ohio State University.

Taylor Flaherty, University of Nevada Las Vegas.

Alexandra Kralick, University of Pennsylvania.

Sterling Wright, The Pennsylvania State University.

